# Chimeric Pathogen-Associated Molecular Patterns (PAMPs) as Vaccine Adjuvants

**DOI:** 10.3390/vaccines14060525

**Published:** 2026-06-12

**Authors:** Bethany M. Potter, Anya F. Weth, Emma M. Dangerfield, Mattie S. M. Timmer, Bridget L. Stocker

**Affiliations:** 1 School of Chemical and Physical Sciences, Victoria University of Wellington, Wellington P.O. Box 600, New Zealand; 2 Department of Chemistry, University of York, York YO10 5DD, UK

**Keywords:** PAMP, PRR, conjugate, TLR, NOD2, Mincle, STING

## Abstract

The development of pathogen-associated molecular patterns (PAMPs) that signal via pathogen recognition receptors (PRRs) on innate immune cells is a strategy that is widely adopted in adjuvant research. Less well studied is how covalently linking different PAMPs affects the immune response. Herein, we summarise the research on the effect of PAMP linkage on innate and adaptive immune responses. These covalently linked or “chimeric” PAMPs often lead to immune cell synergies that are greater than those exhibited by the admixed (unconjugated) PAMPs, with several PAMP conjugates exhibiting remarkable adjuvant activity in a variety of disease contexts that include infectious disease, allergy, and cancer immunotherapy. This improvement in immune cell activation is thought to be due to more effective crosstalk between the different PRR signalling pathways, as conjugation ensures that each cell receives each class of PAMP. In addition, PAMP conjugates can form particulates, which has been postulated to lead to improved adjuvanticity, or they may facilitate the targeting of endosomal PRRs via the PRR-mediated endocytosis of the alternative PAMP in the conjugate. PAMP conjugates can also reduce the toxicity of individual PAMPs. However, not all PAMP conjugates are effective, and there are still many aspects of this research platform that are poorly understood, including how linker chemistry affects the immune response and how PRR signalling pathways or PAMP combinations combine to skew the immune response. We will address these and other outstanding questions that relate to the use of PAMP conjugates as vaccine adjuvants.

## 1. Pathogen-Associated Molecular Patterns (PAMPs) as Adjuvants

### 1.1. An Introduction to Vaccines and Why We Need Conjugated PAMPs as Vaccine Adjuvants

Vaccines play an essential role in reducing the morbidity and/or mortality associated with infectious diseases. They require an antigen, which is an immunogenic protein or carbohydrate antigen that leads to the production of antibodies, and most vaccines require an adjuvant, which enhances and moderates the intensity and type of immune response [1,2,3]. The need for adjuvants to be added to vaccines has become all the more prevalent with the shift towards subunit vaccines that incorporate selected target antigens. The use of specific antigens can limit potential adverse effects of the vaccine but may also decrease vaccine efficacy because of the absence of other immunostimulatory molecules that would be present in whole-cell vaccines [4,5]. Accordingly, adjuvants can be added to improve vaccine efficacy in a more controlled way.

There are many different types of adjuvants, which can be classified according to their main mechanism of action, as briefly discussed below; however, herein, we will focus on the use of pathogen-associated molecular patterns (PAMPs), which target pathogen recognition receptors (PRRs) on innate immune cells [3]. Immunostimulant adjuvants, such as PAMPs, bridge non-specific and specific immunity and augment the immune response against the target antigen [3,6,7]. Notably, the conjugation of PAMPs that target different PRRs can lead to immune cell synergy through the activation of multiple PRR signalling pathways (Section 1.2 and Section 1.3) and has thus been the focus of many research programmes. Here, PAMP conjugates are often compared to their unconjugated (admixed) counterparts for their ability to modulate the immune response to select lead adjuvants (Section 2, Section 3 and Section 4), with application as vaccine adjuvants then being undertaken where appropriate.

Other types of adjuvants include delivery systems (e.g., mineral salts, emulsions or microparticles), immune potentiators (e.g., Toll-like receptor (TLR) agonists), combined adjuvants (e.g., AS01 and AS04), or mucosal adjuvants (e.g., cholera toxin) [3]. In general, delivery system adjuvants, such as alum and lipid nanoparticles, prolong antigen availability and improve antigen uptake by antigen-presenting cells (APCs) [3,5]. Mucosal adjuvants are designed to enhance immunity at the mucosal surfaces (e.g., the gastrointestinal, urogenital and respiratory tracts). Here, the generation of antigen-specific IgA and tissue-resident memory T (T_RM_) and B (B_RM_) cells in mucosal compartments is a key measure of vaccine efficacy, as these cellular mediators are essential for pathogen clearance [8]. Given the focus of this review, the reader is referred to several excellent reviews for further insight into the different classes of vaccine adjuvants [3,8,9,10].

### 1.2. The Different Classes of Pattern Recognition Receptors

Upon binding to their respective PRR, PAMPs instigate complex downstream signalling pathways to express genes associated with cytokine and chemokine production [6]. This, in turn, attracts other immune cells to the site of infection/vaccination and, depending on the PRR(s) activated and the type of PAMP used, skews naïve T cells to a particular T-helper (T_H_) cell subset. There are three main populations of T_H_ cells: T_H_1 cells, which secrete pro-inflammatory cytokines such as IFN-γ, IL-2 and IL-12 to activate macrophages to destroy phagocytised pathogens, or to destroy infected cells themselves [11]; T_H_2 cells, which secrete IL-4, IL-5 and IL-13, leading to the upregulation of antibodies, or IL-10, leading to an anti-inflammatory effect [11]; and T_H_17 cells, which are less well characterised but are associated with the control of extracellular bacteria and fungi via the secretion of IL-17 [12]. While activating any one PRR signalling pathway tends to promote a certain type of immune response, variations in PAMP structure and number greatly influence this. Insofar, different PAMPs can cause the host to respond in a highly pathogen-specific manner even if the PAMPs trigger the same PRRs.

PRRs are typically classified according to their protein domain homology. Membrane-bound PRRs include TLRs and C-type lectin receptors (CLRs), while cytosolic PRRs include nucleotide-binding and oligomerisation domain (NOD)-like receptors (NLRs), retinoic acid-inducible gene-I (RIG-I)-like receptors (RLRs), and absent in melanoma-2-like (AIM2)-like receptors (ALRs), whereby the latter can also be grouped as part of a broader PRR subset of cytoplasmic DNA sensors that includes the stimulator of interferon genes (STING) [6,7]. The classification of other PRRs is more varied; however, two more common subclasses of PRR include inhibitory PRRs (e.g., the Siglecs) and extracellular soluble pattern recognition molecules (PRMs) (e.g., ficolins, collectins, pentraxins) [6]. Many PRRs have been explored as targets for adjuvant development [13], particularly the TLRs [6]. Most notably, the TLR9 agonist CpG 1018 is used in hepatitis B vaccines (HEPLISAV^TM^) [14], while the combined clinical adjuvants AS01 and AS04 contain the TLR4 agonist monophosphoryl lipid A (MPL) in combination with other immunostimulatory molecules (e.g., MPLA and QS-21 for Herpes Zoster, Malaria, and respiratory syncytial virus vaccines) and MPL and Alum for human papillomavirus and hepatitis B vaccines [9].

### 1.3. Conjugating Pathogen-Associated Molecular Patterns Can Augment Cellular Responses

The main sentential cells of the innate immune response [epithelial cells, phagocytic cells and dendritic (DC) cell subsets] simultaneously express overlapping but not identical combinations of PRRs [6]. This allows the cells to induce an effective and highly tailored immune response against invading microorganisms via the activation of several downstream pathways and the concomitant increase or decrease in gene expression [5,6,15,16,17,18]. Accordingly, signalling by multiple PAMPs can lead to additive or even synergistic immune responses (e.g., cytokine responses being greater than that elicited by the combination of the individual PAMPs), and/or subsets of genes may be downregulated [5,6,16,18].

**Figure 1:**
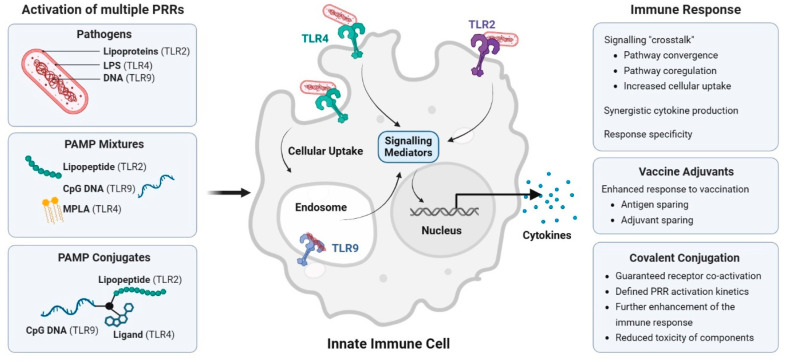
Stimulation of multiple PRR signalling pathways can enhance the immune response. Multiple PAMPs can be delivered to immune cells as part of native pathogens (e.g., TLR2, 4, and 9 ligands in *Streptococcus pneumoniae* and *Mycobacterium tuberculosis*), in mixtures, or via covalent conjugation. Activation of multiple PRRs can induce signalling “crosstalk”, involving pathway convergence and coregulation, increase cellular uptake, lead to synergistic cytokine production, and aid in specificity of the immune response. When used as vaccine adjuvants, multiple PAMPs can enhance the response to vaccination and therefore lead to antigen and adjuvant sparing. Covalent conjugation of PAMPs guarantees that PAMPs reach the same cell, which further augments the immune response and can increase the safety profile of the vaccine.

The observation that the engagement of multiple PRRs can lead to immune cell synergy and a more tailored immune response has led to efforts to recapitulate this in adjuvant development [Figure 1]. In addition to immune cell synergy, vaccines using more than one PAMP can lead to antigen and adjuvant sparing, so less antigen/adjuvant is needed to induce an adaptive immune response [16,19]. This, in turn, can improve the safety profile of the vaccine. There are several strategies that can be used to present multiple PAMP adjuvants to the immune system; however, in general, these fall into one of two categories: the first is via the “admix” addition of individual PAMPs as part of a formulated adjuvant delivery systems; the second is the delivery of chemically conjugated PAMPs in the form of covalently linked dimers, trimers, and, in theory, other polymeric species. Despite the additional synthetic effort that is required to prepare PAMP-PAMP conjugates, chemically conjugating different PAMPs has the advantage in that it ensures the delivery of different PAMPs to the same cell. This facilitates crosstalk between the adjuvants, which can further augment the immune response [6,16,20,21,22,23]. Conjugation can also improve the bioavailability of the individual PAMPs and/or allow for the use of PAMPs that may otherwise exhibit toxicity when administered as individual compounds. For example, the NOD2 agonist MDP leads to systemic inflammation when injected or taken orally [24] but can be successfully administered as part of a PAMP chimera (Section 3.3.2, Section 3.3.3 and Section 4.3.2).

## 2. Conjugated Toll-Like Receptor (TLR)-TLR Agonists as Vaccine Adjuvants

### 2.1. TLR Signalling Pathways

**Figure 2:**
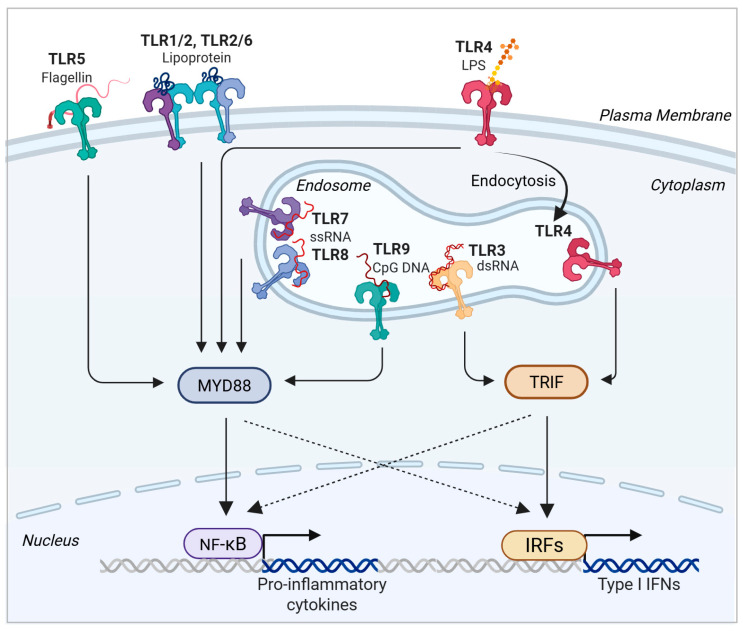
Cellular location, ligands, and signalling pathways of TLRs. Extracellular TLRs 1, 2 and 6, TLR4, and TLR5 and intracellular TLRs 7, 8 and 9 signal through MyD88. MyD88 signalling results in the activation and translocation of NF-κB to the nucleus and the production of pro-inflammatory cytokines. Activation of TLR4 leads to endocytosis and, along with TLR3, signalling through TRIF and the activation of the IRF pathway, leading to type I interferon production. Dotted arrows indicate that MyD88 signalling can also contribute to IRF activation, and activation of the TRIF pathway can also contribute to NF-κB activation.

TLRs are the most extensively studied and well-characterised PRRs. They are found both extracellularly (TLRs 1, 2, 4, 5 and 6) and in endosomes (TLRs 3, 7, 8 and 9) of APCs [6,25,26] and cooperate to affect innate immune cell responses [5,22,25,26,27]. The cellular location of a TLR reflects the origin of the ligand that it is responsible for detecting, with the extracellular TLRs recognising lipoproteins (TLR1, 2 and 6), lipopolysaccharide (LPS) (TLR4), and bacterial flagellin (TLR5) from the extracellular compartments of pathogens, and the intracellular TLRs recognising intracellular molecules from pathogens such as double-stranded RNA (dsRNA, TLR3), single-stranded RNA (ssRNA, TLR7 and 8), and unmethylated cytosine–phosphate–guanine (CpG) DNA (TLR9) [28] [Figure 2]. Endosomal TLR4 also recognises LPS in endosomes, which has recently been shown to be an event that can be independent of surface-expressed TLR4 signalling [29]. The binding of PAMPs to TLRs induces homo- or heterodimerization of TLR receptors and the activation of subsequent downstream signalling pathways [25,26,27]. Of the TLRs, all except TLR3 share a common signalling pathway using myeloid differentiation factor 88 (MyD88), which causes downstream signalling to activate nuclear factor-κB (NF-κB) transcription factors as the major signalling pathway and the release of pro-inflammatory cytokines, including interleukins (ILs), thereby augmenting the T_H_1 immune response [6,25,26,27]. In contrast, TLR3 and endosomal TLR4 use the Toll/interleukin-1 receptor/resistance protein (TIR) domain-containing adaptor inducing IFN-β (TRIF)-dependent pathway. This results in the expression of interferon regulatory factors (IRFs) and subsequent production of type 1 interferons, which are important for host defence against viruses [30]. However, the two pathways overlap as MyD88 can trigger IRFs for antiviral defence, and TRIF can trigger NF-κB to sustain the inflammatory response [26,31].

TLR10 is the latest human TLR to be discovered. Its function and ligands are still poorly defined, although TLR10 is thought to have an anti-inflammatory effect by dimerising with TLR1, TLR2 and TLR6 [32]. However, the effects of PRR signalling can vary, depending on the exact ligand that binds. For example, although the heterodimerization of TLR2 with TLR1 or TLR6 is predominantly associated with an inflammatory response, in *Yersinia pestis* [33] or *Mycobacterium tuberculosis* [34] infection, TLR2/6 dimerization leads to IL-10 production and a dampening of the immune response.

### 2.2. Common TLR Ligands Used in PAMP-PAMP Chimeras

There are many examples to illustrate that the simultaneous targeting of multiple PAMPs can lead to immune cell synergy [5,21,27,35,36]. Indeed, the ability of the live attenuated yellow fever vaccine to activate multiple DC subsets via TLR2, 7, 8 and 9 is thought to contribute to the vaccine’s remarkable efficacy [37]. It is therefore not surprising that many studies have been undertaken into the effect of conjugated TLR agonists as vaccine adjuvants.

**Figure 3:**
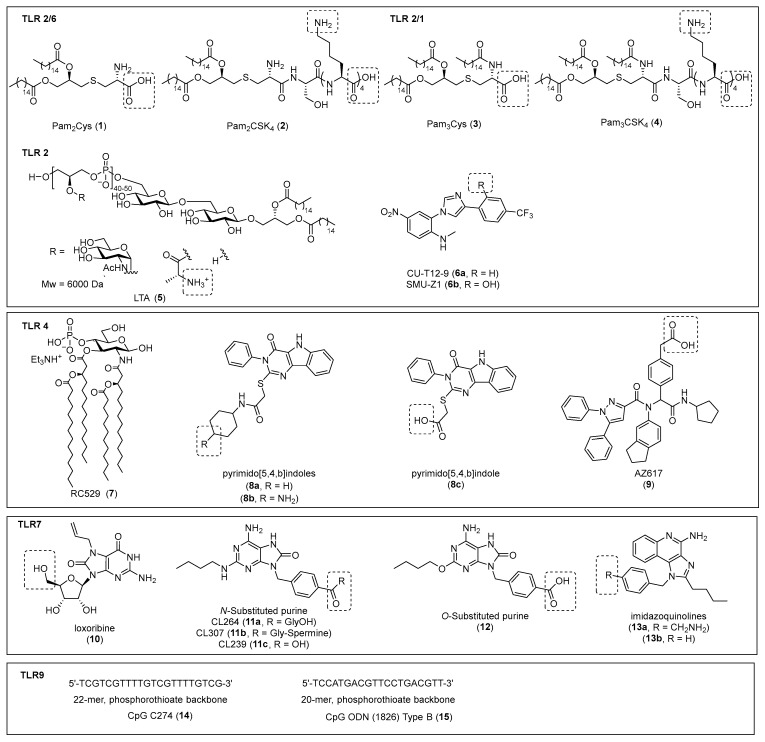
Structures of TLR ligands used in PAMP-PAMP conjugates. Sites that might be useful for conjugation are highlighted using dotted boxes.

Four common PAMPs used for chimeric adjuvants are the TLR2 ligands Pam_2_Cys (**1**) and Pam_2_CSK_4_ (**2**) and their trilipid counterparts Pam_3_Cys (**3**) and Pam_3_CSK_4_ (**4**), which contain the peptide moiety SK_4_ (serine–lysine–lysine–lysine–lysine) to enhance agonist activity and solubility [38] [Figure 3]. Both classes of ligands bind TLR2 via two ester-bound palmitoyl chains, with *R*-enantiomers exhibiting greater potency as adjuvants [39,40,41]. Since Pam_2_Cys and Pam_2_CSK_4_ do not have an acyl group at the *N*-terminus, they rely on the head group of the ligand for TLR2/6 dimerization [42]. In contrast, Pam_3_Cys and Pam_3_CSK_4_ contain an acyl chain that is inserted into a lipophilic pocket in TLR1 to facilitate TLR2/1 dimerization [41]. There are no TLR2/6 interactions with any of the four lysine residues of Pam_2_CSK_4_ (**2**). Similarly, the side chains of the four lysine residues of Pam_3_CSK_4_ (**4**) also have limited interaction with TLR1 and TLR2, except for a hydrogen bond of Lys4″ with Asn294 of TLR2 [41]. Accordingly, conjugation of Pam_2_CSK_4_ (**2**) or Pam_3_CSK_4_ (**4**) to another biomolecule or linker can occur at a lysine side chain, although often coupling occurs at the peptide *C-*terminus as functionalisation at this site is less likely to perturb receptor–ligand interactions.

Another commonly used TLR2 ligand for PAMP conjugates is lipoteichoic acid (LTA, **5**). Structural differences amongst LTA derivatives have been observed, although it is generally thought that the two lipid chains of LTA are inserted into the binding pocket of TLR2 in a similar fashion to Pam_2_CSK and Pam_3_CSK [43]. Insofar, the hydrophilic sugars and repeating units of LTA interact with TLR6 or TLR1 to form the corresponding TLR heterodimer. The primary amine of the d-alanine side chain in LTA is the most favourable site for conjugation. More recently, the small-molecule TLR2/1 agonist CU-T12-9 (**6a**) [44] and its 2-OH derivative SMU-Z1 (**6b**), which possess notable adjuvant properties, particularly for the suppression of leukaemia [45], were used in the development of several classes of PAMP chimeras (Section 2.4.2 and Section 2.4.3). Both agonists (**6a**) and (**6b**) are potent TLR2/1 agonists, and the hydroxyl function in **6b** was conveniently used as the linkage site for conjugate formation.

TLR4 forms a complex with the myeloid differentiation factor (MD-2) upon ligand binding [46], with the main native ligand for TLR4 being lipopolysaccharide (LPS), a major component of Gram-negative bacteria. The acyl chains of lipid A in LPS are inserted deep into the MD-2 pocket, while the two phosphate groups of the carbohydrate moiety form charge and hydrogen bond interactions with charged residues in MD-2 and TLR4 [46]. LPS is too toxic for use in humans; however, structure–activity relationship (SAR) studies led to the identification of monophosphoryl lipid A (MPLA), which is less toxic and now a clinically used vaccine adjuvant [47]. MPLA binds to TLR4 via its hydrophobic lipids, leaving the exposed functional groups of the sugar motifs as potential handles for conjugation [47]. Studies suggest that the nonreducing 6′-hydroxyl conjugates of MPLA have superior immunological activity compared to the 1-*O*-conjugates [48]; however, to date, MPLA is yet to be used in PAMP-PAMP conjugates. This is likely due to its heterogeneity, high cost and complex synthesis. Accordingly, less complex but still potent TLR4 glycolipid agonists, such as RC529 (**7**) (also known as Ribi529) [49], have been used for PAMP-PAMP conjugates.

Several small-molecule TLR4 agonists have been identified over the years as part of an ongoing search for more readily accessible PRR ligands [50]. Because the binding of small molecules to TLR4 is not always known, the choice of TLR4 agonist and the conjugation site for PAMP chimeras is often based on SAR studies using the parent compound and/or the ease of chemical synthesis. Several pyrimido[5,4,*b*]indole derivatives have been used in PAMP conjugates, including pyrimido[5,4,*b*]indoles **8a** and **8b**, which were identified from the parent series [51,52] and subsequently equipped with a chemical handle on the acyl group (**8c**) for conjugation. Molecular modelling of the pyrimido[5,4,*b*]indoles into the TLR4/MD-2 complex suggests that the acyl chain that is connected to the 2-position of the pyrimido[5,4,*b*]indole scaffold (via a -SCH_2_-linkage) forms strong interactions with MD-2 [51]. This brings into question the choice of this site for conjugation, although synthetically, it is more accessible than the 9-position, which is likely more solvent-exposed within the TLR4 binding pocket. Similarly, AZ617 (**9**) [53] has also been used for the synthesis of a variety of TLR4 PAMP-PAMP conjugates.

The natural ligands for TLR7 and TLR8 are ssRNAs of viral origin [54]; however, the poor drug properties of RNA led to the development of many small-molecule agonists for TLR7 and/or TLR8. Perhaps the most well-known of these is the selective TLR7 agonist imiquimod, which is approved for topical application for the treatment of certain skin conditions (but is too toxic to be administered systemically). The small-molecule TLR7/8 agonists used in PAMP/PAMP conjugates include loxoribine (**10**), *N*-and *O*-substituted purines (**11** and **12**) [55,56], and imidazoquinolines (TLR7/8) **13**. The activation of the innate immune system via loxoribine (**10**) is similar to imiquimod and is largely restricted to TLR7 [57,58]. A crystal structure of monkey TLR7 in complex with loxoribine (**10**) shows the guanine motif bound to the receptor, while the ribose moiety is only loosely recognised by TLR7 with one H-bond between the 5′-OH group and the N of T586 [59]. Notwithstanding, many PAMP conjugates containing loxoribine are functionalised at the N^2^-position of the guanine ring, which is presumably due to synthetic accessibility.

The selective TLR7 ligand CL264 (**11a**) is an alkylated derivative of 9-benzyl-8-hydroxyadenine [60]. It was observed that alkylation on the 2-position of the adenine ring in 9-benzyl-8-hydroxyadenine led to increased IFN production [60], thus making this position suitable for functionalisation with a carboxy linker and subsequent PAMP conjugation. Imidazoquinoline derivative (**13a**) is a methylamine-functionalised derivative of a TLR7/8 agonist (**13b**) that had impressive nanomolar potency (59 nM) against TLR7 [61], with later work reporting a mixed TLR7/8 profile of the parent ligand [62]. Although the original imidazoquinoline **13a** from which **13b** was derived was not the most potent of the series [61], the benzyl group on **13b** allows for conjugation while retaining good immunomodulatory activity, as evidenced by dimers of **13a** that retained good (0.11 µM) TLR7 agonist activity [63].

The last TLR to be targeted in the design of PAMP conjugates is TLR9. TLR9 senses DNA rich in unmethylated CpG dinucleotides and binds synthetic CpG oligodeoxynucleotides (ODNs) (e.g., CpG C274 **14** and CpG ODN 1826 Type B **15**), which are short, single-stranded DNA (ssDNA) oligomers with unmethylated CpG motifs and a phosphorothioate, rather than the native phosphodiester, backbone. The sequence specificity of the CpG ODN dictates the species specificity of the ligand, with further classification into Class A CpG ODNs, Class B ODNs and Class C ODNs defining the effect of the CpG ODN structural motif on human peripheral blood mononuclear cells (hPBMCs) [64]. CpG ODNs have been conjugated to a variety of biomolecules, including antigens, antibodies, lipids, nanoparticles and cancer cells [28,65], as well as to numerous other TLRs (Section 2.4.4, Section 2.4.6 and Section 2.5.2). The crystal structure of CpG conjugated to horse TLR9 suggests that the 5′-end is more amenable to conjugation [66]; however, the 5′ end is positioned towards the endosomal membrane, with some suggesting that this may interfere with the efficacy of 5′-conjugates, particularly when conjugating larger molecules [28]. Notwithstanding, conjugation to both the 3′ and 5′ ends of CpG can yield functional bioconjugates.

In contrast to the TLRs 2, 4, 7/8 and 9, PAMP-PAMP conjugates containing TLR3 and/or TLR5 ligands are yet to be developed. Native TLR3 ligands are dsRNA that bind to TLR3 dimers, with the most commonly employed TLR3 agonist being the high-molecular-weight synthetic dsRNA analogue, polyinosinic–polycytidylic acid (poly(I:C)) [67]. TLR5 recognises flagellin (the principal component of bacterial flagella in both Gram-positive and Gram-negative bacteria), as well as a highly conserved 13-amino acid sequence within the D1 domain of the flagellin monomer [68]. This has spurred the synthesis of flagellin-derived peptides [69] that have been conjugated to antigens [70] as well as to antimicrobial peptides to trigger plant defence responses [71]. Despite this, no flagellin peptide PAMP-PAMP conjugates have been reported.

### 2.3. Chemical Approaches to the Conjugation of PAMPs

A wide range of chemoselective ligation strategies has been used for the synthesis of PAMP-PAMP conjugates. The choice of chemistry is often dictated by the functional groups naturally present on PAMPs and whether preservation of receptor-binding activity is required. Among the available approaches, amide bond formation is widely used. Carboxylic acid and amine functionalities, either already present in the PAMP agonist or introduced through synthetic modification, can be coupled using reagents such as 1-ethyl-3-(3-dimethylaminopropyl)carbodiimide (EDCI)/*N*-hydroxysuccinimide. (NHS), hexafluorophosphate azabenzotriazole tetramethyl uronium HATU, or Benzotriazol-1-yloxytripyrrolidinophosphonium hexafluorophosphate (PyBOP) [72]. Amide linkages are especially attractive because of their robustness and synthetic simplicity, although, as with all conjugation strategies, care must be taken to avoid modifying pharmacophores that are required for receptor binding or inhibiting receptor–agonist interactions through steric congestion. Free amines may also need to be protected and then un-masked prior to coupling to avoid undesirable side reactions.

Bioorthogonal “click” chemistry is another important ligation strategy that has become increasingly important in the construction of PAMP-PAMP conjugates. Copper-catalysed azide–alkyne cycloaddition (CuAAC) enables the rapid and selective formation of stable triazole linkages between azide- and alkyne-functionalised PAMPs [73]. The high yields, functional group tolerance, and modularity of CuAAC make it particularly well suited for the synthesis of highly complex conjugates. Thiol-based ligation methods represent another important class of conjugation reaction, as thiol–maleimide chemistry enables rapid formation of stable thioether bonds under mild conditions [74]. Finally, for carbohydrate-containing PAMPs, chemistries based on aldehyde functionalisation are particularly useful. These include oxime and hydrazone ligations, which occur via the reaction of aldehydes with aminooxy or hydrazide groups, respectively [75,76]. These reactions proceed efficiently in aqueous media and can provide either stable or stimuli-responsive linkages, with oxime bonds typically being more hydrolytically stable than hydrazone bonds, although both will be cleaved under increasingly acidic conditions, such as those within the lysosome [77].

### 2.4. Dimeric TLR Chimeras

#### 2.4.1. Summary of Dimeric TLR Agonist Chimeras Prepared to Date

**Figure 4:**
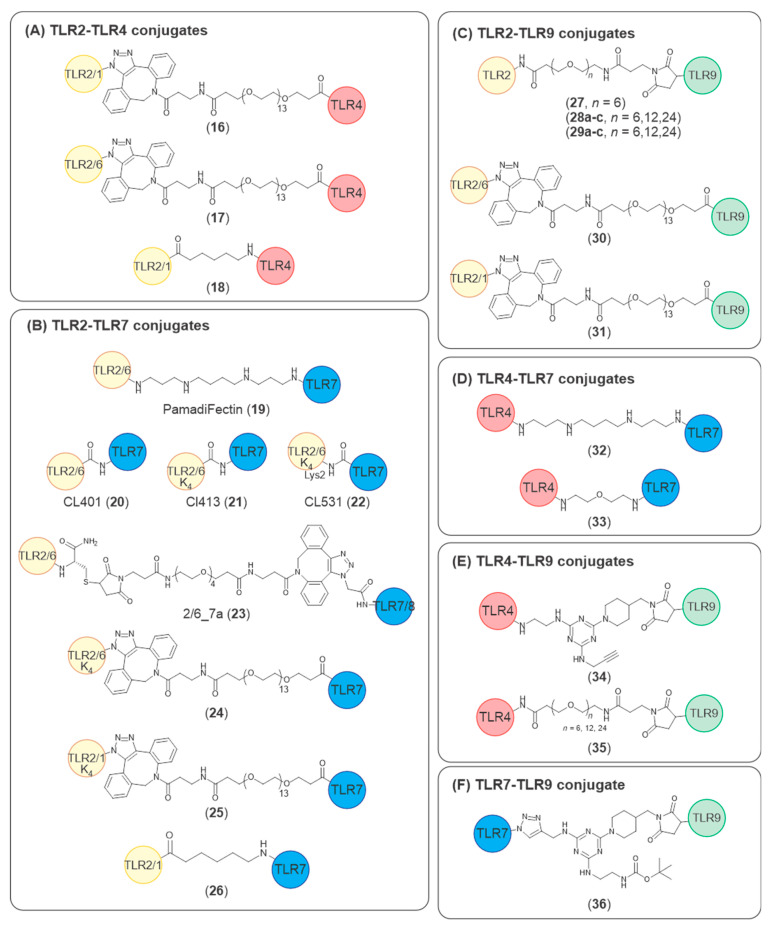
Schematic representations of TLR-TLR agonist conjugates.

Several chimeric PAMP conjugates have been synthesised [Table 1]. These contain ligands for TLR2 or TLR2/6, TLR4, TLR7 and/or TLR9 [19,78,79,80,81,82,83,84], with further details of the compound structures being provided in Figure 4. To date, much of the research into TLR-TLR PAMP conjugates has focussed on conjugation strategies and the effect of spatial orientation on immune cell activation, particularly the activation of macrophages (Mφ) and DCs in vitro. However, some studies investigating the adjuvanticity of PAMP chimeras in combination with an antigen have been performed.

#### 2.4.2. TLR2-TLR4 Agonist Conjugates

To explore whether the conjugation of TLR2 and TLR4 ligands could provide synergistic immune cell activation, Kimani et al. developed two TLR2-TLR4 agonist conjugates (**16** and **17**, Figure 4A), which contained the TLR4 agonist pyrimido[5,4-b]indole (**8b**) and either Pam_2_CSK_4_ (**2**) or Pam_3_CSK_4_ (**4**) as the TLR2 ligand, respectively [78]. These conjugates were constructed using a heterotelechelic polyethylene glycol (PEG) linker equipped with a cyclooctyne for a strain-promoted cycloaddition reaction with azide-functionalised Pam_3_CSK_4_ or Pam_2_CysK_4_, and an *N*-hydroxysuccinimide (NHS)-protected ester for conjugation to the amide-functionalised indole. The administration of the admixed TLR2/1 and TLR4 ligands did not increase NF-κB signalling in murine Mφ, or TNF or IL-6 production by bone marrow-derived macrophages (BMDMs) (25 nM at 24 h), with both responses being similar to those generated by the TLR2 ligand alone [78]. The TLR2/6-TLR4 ligand conjugate (**17**) also led to lower levels of NF-κB signalling and IL-6 production than the admixed ligands; however, greater levels of TNF by BMDMs were observed. To further explore these phenomena, the production of TNF by RAW 264.7 MΦ was measured over 24 h at various ligand concentrations (10, 25 and 50 nM); however, neither of the TLR2-TLR4 ligand conjugates led to TNF production that was better than the admix combination or, indeed, stimulation with Pam_3_CSK_4_. Single-cell NF-κB dynamics revealed that the TLR2/1-TLR4 dimer (**16**) led to poor levels of NF-κB and cytokine production and had a profile that was similar to that elicited by cells stimulated with the indole (TLR4 ligand) alone, thereby suggesting that this dimer preferentially signalled via TLR4 [78]. More recently, Janez et al. also prepared a TLR2/6-TLR4 ligand conjugate (**18**) containing the small-molecule TLR2/1 agonist CU-T12-9 (**6**) and the pyrimido-indole derivative 8c, with this work still to be peer-reviewed at the time of writing this manuscript [79]. However, this combination of PAMPs did not provide enhanced immune activation compared to that observed for other PAMP conjugates.

Taken together, it thus remains to be determined whether TLR2 and TLR4 ligands can be conjugated to lead to immune cell synergy that is greater than admixed ligands. Signalling via these two TLRs will activate two different signalling pathways, with binding of the lipophilic ligands to TLR2 leading to TLR2 dimerization, preferential partitioning and clustering of the TLR2–ligand dimers into plasma membrane microdomains and the instigation of MyD88-dependent signalling [26,27]. In contrast, TLR4 ligands engage the Lipopolysaccharide Binding Protein (LBP)/CD14/MD-2 axis to access both plasma membrane- and endosome-associated signalling pathways, thereby contributing to TRIF-dependent outputs [30]. Accordingly, unlike many other chimeric PAMP conjugates that pair ligands for extracellular and intracellular PRRs and lead to enhanced immunity, presumably by enhancing the uptake of the intracellular PAMP (vide infra), the TLR2-TLR4 PAMP-PAMP combination needs to be optimally arranged to engage two extracellular TRL2/TLR4 receptors to allow for receptor dimerization and also receptor clustering. In theory, this could be achieved, but based on the data at hand, it appears to be more difficult to realise. It is also possible that a fixed 1:1 stoichiometry of the TLR2-TLR4 PAMP conjugate is insufficient for receptor clustering, or that internalisation of the TLR4–ligand complex into the endosome may be hindered if the complex is also tethered to a TLR2/ligand pair. Further work is required to address these issues.

#### 2.4.3. TLR2-TLR7 Agonist Conjugates

Conjugation of TLR2 and TLR7 agonists can induce synergistic T_H_1-skewed immunomodulatory effects in vitro and in vivo, which do not occur with the coadministered PAMPs [80,81]. PamadiFectin (**19**, Figure 4B) contains Pam_2_Cys (**1**) connected at its *C*-terminus to the hydroxy-adenine TLR7 agonist CL307 (**11b**), a spermine-containing analogue of CL264 (**11a**), and was found to induce synergistic DC activation and gene transcription for cytokines associated with both T_H_1 and T_H_2 polarisation [80]. No such synergistic increase was observed for the admixed ligands. PamadiFectin (**19**) also primed CD8+ T cells, although there was no significant difference between the conjugate or admixed ligands. Notably, combining PamadiFectin (**19**) with a nanoparticle formulation containing the HIV-1 p24 antigen significantly enhanced antigen-specific IgG titres one week after vaccination, with the adjuvanticity of PamadiFectin (**19**) being maintained after 14 days (3 vaccinations). Although antibody titres at week three were similar to those elicited by the individually administered PAMPs, the avidity of the antibodies was greater when using PamadiFectin (**19**), and the immune response exhibited a more balanced T_H_1/T_H_2 response. Moreover, systemic toxicity was associated with the individual PAMPs but not the conjugate. Mechanistically, the authors proposed that the strong T_H_1/T_H_2 profile of the conjugate was due to its self-assembling nanoparticle structure and ability to engage TLR2 (thereby favouring T_H_2 immunity). This TLR2 engagement also enhanced ligand endocytosis, subsequent engagement with TLR7, and, thus, induction of a T_H_1 immune response.

Shortly following the studies by Gutjahr et al. [79], Laiño et al. synthesised three TLR2-TLR agonist conjugates: CL401 (**20**), which contains Pam_2_Cys (**1**) linked via an amide to CL264 (**11a**); CL413 (**21**) containing the TLR2/6 agonistPam_2_CSK_4_ (**2**) conjugated at its carboxy terminus to CL2764 (**11a**); and CL531 (**22**) containing the TLR2/6 agonist Pam_2_CSK_4_ (**2**), which was connected via its second lysine residue to TLR7 agonist CL264 (**11a**) [81,85]. Across various concentrations, all conjugates typically led to a reduction in IL-1β by DCs in vitro compared to the admix ligands, and CL413 (**21**) resulted in a modest increase in IL-6 production; however, both CL513 (**22**) and CL413 (**21**) led to a significant increase in IL-10 compared to the individual PAMPs or admix combinations, which indicated their ability to skew the immune response to a T_H_2-phenotype [81]. Indeed, this was realised when CL513 (**22**) and CL413 (**21**) were found to suppress OVA-induced T_H_2 secretion in a mixed lymphocyte assay (significant decrease in IL-13 and IL-5). CL401 (**20**) enhanced OVA-induced IL-17A secretion. With a view to using the conjugates in vaccinations to mitigate allergic reactions, the ability of CL513 (**22**), CL413 (**21**) and CL401 (**20**) to induce mast cell activation and degranulation was then investigated in vitro. All ligand combinations (except the TLR7 agonist alone) led to limited mast cell activation with very little degranulation (which would be indicative of an allergic response). The in vivo administration of the conjugates in mice and the measurement of cytokines revealed that CL513 (**22**) and CL413 (**21**) led to an increase in IL-6 and TNF (though less than the admix combination), while CL401 (**20**) did not lead to cytokine production. Conjugate CL513 (**22**) was then used as a prophylactic vaccine in a mouse model of OVA-induced intestinal allergy, where it was found to increase IgG1 (though to similar levels as OVA + the individual PAMPs). However, CL513 (**22**) significantly increased IgG2a, decreased IgE production and decreased β-hexosaminidase release (an indicator of mast cell degranulation/allergic response) with respect to all groups, thereby providing support for its use as an adjuvant in vaccines for reducing allergic responses. In other studies, it was determined that TLR2 engagement was observed when exploring the efficacy of CL401 (**20**), CL413 (**21**) and CL531 (**22**) in the context of anticancer immunotherapies [85]. It was hypothesised that the three-dimensional structure of the dual TLR2/7 ligands is a key factor for their immunomodulatory effect, with both CL413 (**21**) and CL531 (**22**) adopting structures that allow for interactions with TLR2 on the cell surface, which, in turn, promotes internalisation and TLR7 interaction.

In 2020, Manna et al. prepared a TLR2/6-TLR7/8 ligand conjugate 2/6_7a (**23**) containing PAMPs Pam_2_CSK_4_ (**2**) and imidazoquinoline (**13a**) for TLR2/6 and TLR7/8, respectively [82]. These were linked by a short peptide and combined with a carbohydrate polymer to form nanoparticles that were designed to prevent the rapid and systemic diffusion of the PAMP agonists and improve the therapeutic window for anticancer therapies [82]. As part of their initial investigations, the abilities of the different constructs to activate RAW 264.7 Mφ were determined, where it was found that conjugate 2/6_7a (**23**) and the admixed PAMPs led to similar levels of NF-κB activation and IL-12p70. However, the conjugate resulted in higher levels of IFN-β production, and across all readouts, the multicomponent aggregate typically performed better, including in antitumour experiments using a B16.F10 tumour challenge model. Here, enhanced CD8 and Natural Killer (NK) cell-mediated antitumour responses, as well as ultralow off-target toxicity, were observed using the aggregate. Compared to the admixed PAMPs, the conjugate alone led to better antitumour immunity, though this was significantly less than that exhibited by the chimeric PAMP containing nanomaterial. Given this promising result, it would be interesting to explore how the inclusion of PAMP conjugates into nanoparticle delivery systems affects prophylactic vaccines for infectious disease.

Kimani et al. also investigated the efficacy of TLR2-TLR7 agonist conjugates to alter cytokine responses in vitro [78]. Murine Mφ were activated by PAMP conjugates **24** and **25** that contained Pam_2_CSK_4_ (**2**) or Pam_3_CSK_4_ (**4**) conjugated (via their terminal lysine and a PEG linker) to the TLR7/8 ligand imidazoquinoline (**13a**), respectively [78]. Compared to the admixed PAMPs, the conjugates led to decreased NF-κB signalling and no increase in TNF or IL-6 production (indeed, the conjugate containing Pam_3_CSK_4_ (**4**) led to a significant decrease in TNF and IL-6). In studies that are still to be peer-reviewed, Janez et al. also made a TLR2/1–TLR7 agonist conjugate (**26**) containing the small-molecule agonists CU-T12-9 (**6**) and purine (**12**) [79]. This particular PAMP combination showed enhanced cytokine release from human PBMCs and enhanced PBMC cytotoxicity compared to the admixed agonists.

#### 2.4.4. TLR2-TLR9 Agonist Conjugates

Several TLR2/9 ligand conjugates have been synthesised containing CpG (TLR9 agonist) conjugated to LTA (**5**), Pam_2_CSK_4_ (**2**) or Pam_3_CSK_4_ (**4**) (Figure 4C) [78,83,84]. The first of these studies involved the use of a PEG-based linker containing an NHS ester for conjugation to LTA (**5**) (via amide formation) and a maleimide moiety for conjugation to the 3′-end of a thiol-functionalised CpG 1826 (**15**) to yield conjugate **27** [83]. The conjugate significantly increased NF-κB signalling in murine Mφ and activated BMDCs to a greater extent than the admixed ligands (e.g., an increase in CD40 by >40%, and an increase in TNFα, IL-6 and IL-12 by >30%). Using a series of TLR2 and TLR9 antagonists and/or inhibitors, it was determined that the stimulation of Mφ proceeded via the TLR2/6 and TLR9 pathways, although another pathway was also potentially activated. Taken together, this data provides the first evidence that chimeric TLR2-TLR9 PAMPs can lead to immune-mediated synergies.

The same group then undertook further studies to examine the effect of linker length on immune cell activation [84]. For the TLR2-TLR9 ligand conjugates containing LTA (**5**) as the TLR2 ligand (conjugates **28a**–**c** and **29a**–**c**), the conjugates led to increased Mφ NF-κB signalling, with this response being greater for the longer-length linkers. The production of IL-6 by BMDCs was also greater for the conjugates; however, the effect of linker length was variable. In contrast, the TLR2-TLR9 ligand conjugate containing Pam_2_CSK_4_ (**2**) (linked via a thiol on an additional cysteine residue) did not augment NF-κB signalling or IL-6 production; rather, a slight decrease in activity by the C12-linked conjugate was observed. Using TLR2 knockout BMDCs, it was postulated that the larger steric bulk of the linkers hindered the ability of the linked CpG motif to activate TLR9 and that the larger LTA motif had more of a disruptive effect on CpG-induced TLR9 activation compared to the Pam_2_CSK_4_ TLR2 ligand [84], although it should be remembered that some good immune cell activation was observed by LTA-CpG conjugate **27** [83]. In related work, Kimani et al. produced a Pam_2_CSK_4_–CpG conjugate (**30**), which, when used at 50 nM, led to an increase in NF-κB signalling by Mφ compared to the other groups (individual or admixed PAMPs) [78]. However, no enhancement in cytokine production by DCs was observed at any concentration. In contrast, a Pam_3_CSK_4_–CpG conjugate (**31**) led to an increase in NF-κB signalling by Mφ compared to the individual PAMPs and Pam_3_CSK_4_ + CpG, and, when used at a lower concentration (10 nM), resulted in increased TNF production by DCs, particularly at earlier time points [78]. Taken together, this data demonstrates how subtle changes in the TLR2-TLR7 agonist combinations, including not only their type but also linkage, can influence immune-modulating parameters.

#### 2.4.5. TLR4-TLR7 Conjugates

The pyrimido-indole (**8c**) (TLR4) and loxoribine (**10**) (TLR7) conjugate (**32**) was prepared as part of a larger study investigating the effects of trimeric conjugates on immune cell activation (Figure 4D) [19]. The indole-lox dimer (**32**) exhibited little to no NF-κB signalling (RAW264.7 Mφ), IL-12 (BMDCs), and gave a very similar gene transcription profile to resting BMDCs. This was attributed to the lower potency of loxoribine [19]. More recently, Janez et al. synthesised a TLR4/TLR7 ligand conjugate that contained the pyrimido-indole **8c** and the purine-based TLR7 agonist **12** that were linked via a bis(2-aminoethyl)ether spacer [79]. Although this work was yet to be peer-reviewed at the time of writing, the TLR4/TLR7 ligand conjugate (**33**) exhibited broad immune activation in vitro as well as enhanced antigen-specific cellular and humoral responses in mice, with robust antitumour responses in a syngeneic mouse B16F10 tumour model.

#### 2.4.6. TLR4-TLR9 Agonist Conjugates

Two TLR4-TLR9 ligand conjugates have been made, both containing pyrimido-indole (**8**) (TLR4) and CpG (**15**) (TLR9) PAMPs (Figure 4E) [19,84]. The first pyrimido-indole_CpG conjugate (**34**) was developed as part of a study investigating the effects of three PAMPs on immune cell activation, whereby the third ligand-binding site on the functionalised triazine scaffold was kept as the alkyne, resulting in dimeric conjugate (**34**) [19]. In this study, it was demonstrated that the pyrimido-indole_CpG conjugate (**34**) initiated significant NF-κB activation (in a RAW-Blue 264.7 macrophage cell line) and IL-12 production by BMDCs. Although the changes in gene transcription in response to the indole_CpG conjugate (**34**) were less than those exhibited by an indole_loxoribine_CpG trimer (see Section 2.5.2), the conjugation of the indole to CpG did increase CpG/TLR9 activation and decrease the transcription of a subset of inflammatory genes.

The next TLR4_TLR9 agonist conjugate (**35**) synthesised contained a PEG linker between indole (**8b**) and CpG (**15**) and, in addition to exploring the immune-stimulating properties of the conjugated versus the nonconjugated PAMPs, investigations into the effect of linker length on immune cell activation were also undertaken [84]. Here, conjugation of the two PAMPs significantly increased NF-κB signalling by Mφ for all linker lengths when compared to the unconjugated PAMPs (most notably for the C12 length linker), while IL-6 production by BMDCs was significant compared to the admixed ligands for the C6 and C12 linkers only. Using TLR4^−/−^ and TLR9^−/−^ BMDMs, it was determined that CpG signalling was less negatively affected by conjugation to the indole compared to CpG signalling with conjugates containing CpG and either LTA or Pam_2_CSK_4_C. This effect was attributed to the small size of the indole moiety relative to the other two larger PAMPs (LTA or Pam_2_CSK_4_C) [84].

#### 2.4.7. TLR7-TLR9 Agonist Conjugates

The final type of dimeric TLR PAMP conjugate that was explored was that containing loxoribine (**10**) (TLR7) and CpG (**15**) (TLR9) (Figure 4F) [19]. This work was undertaken as part of comparative studies on trimeric PAMPs, whereby the third amine conjugation handle on the triazine scaffold was capped with a Boc group for the synthesis of the Lox_CpG chimera (**36**) [83]. Chimera **36** led to NF-κB signalling in a HEK-Blue 264.7 macrophage cell line and IL-12 production and an overall increase in gene transcription by DCs, although these responses were less than those exhibited by the indole_CpG conjugate (**34**) and similar to those exhibited by the CpG core alone. Insofar, the authors suggested that the low immunostimulatory properties of loxoribine (**10**) led to little overall improvement in immune cell modulation.

### 2.5. Trimeric TLR Agonist Conjugates

#### 2.5.1. Summary of Trimeric TLR Agonist Conjugates Prepared to Date

Trimeric TLR agonist conjugates have been prepared by conjugating PAMPs to a molecular core with three different functionalities for orthogonal linkage [19,86]. The combinations of ligands used to date involve similar combinations to those used for the dimeric TLR PAMP conjugates: namely, ligands for TLR2 [Pam_2_CSK_4_ (**2**) or Pam_3_CSK_4_ (**4**)], TLR4 [pyrimido-indole (**8**)], TLR7 [loxoribine (**10**)], TLR7/8 [imidazoquinoline (**13a**)], and/or TLR9 (CpG, **15**) [Table 2]. In general, the trimeric conjugates skewed the immune response towards the activity of the most potent conjugated agonist; however, the ensuing immune profiles were distinct from the unlinked mixtures and depended on the exact ligand combination [19,86,87]. For example, the use of the TLR9 ligand CpG led to more of a T_H_1 bias, while incorporation of the TLR2/6 agonist Pam_2_CSK_4_ skewed more towards a T_H_2 bias for trimeric conjugates that also contained CpG [80]. Other factors to keep in mind include the observation that subtle changes to the linkers can lead to different T_H_-skewing profiles [19,86].

#### 2.5.2. Molecular Details of TLR-PAMP Trimeric Agonist Conjugates

**Figure 5:**
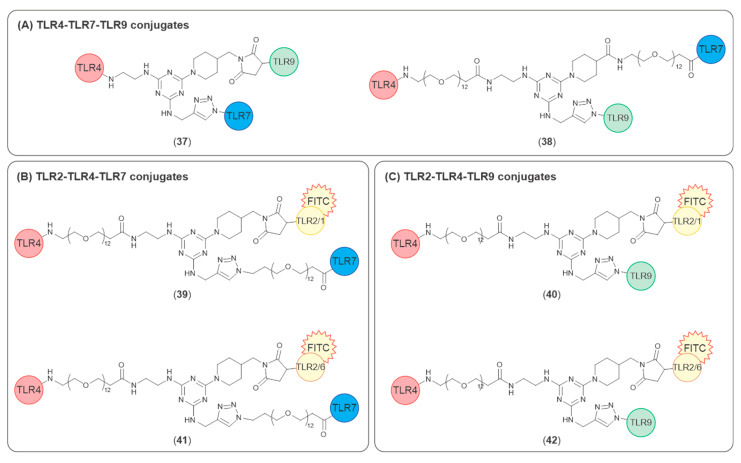
Schematic representation of trimeric TLR agonist conjugates.

The pyrimido-indole/loxoribine/CpG conjugate (**37**) [TLR4_7_9] was the first trimeric PAMP synthesised (Figure 5) [19]. The individual PAMPs were conjugated to a triazine core containing an alkyne (for a copper-catalysed Huisgen cycloaddition reaction), an amine (for amide bond formation), and a maleimide (for a thiol Michael addition). RAW264.7 Mφ treated with the indole–loxoribine_CpG conjugate (**37**) led to an increase in NF-κB signalling compared to all other groups except the indole–CpG dimer (**34**). The similarity between the trimer (**37**) and dimer (**34**) was attributed to the comparatively lower immunostimulatory activating of loxoribine, as previously noted (Section 2.4.5). However, the inclusion of loxoribine in the trimer did lead to an increase in overall gene transcription levels by BMDCs for both T_H_1 and T_H_2 responses, which was greater than that elicited by all other groups. A small subset of genes associated with TNF and inflammation were also downregulated in response to the trimer. Taken together, this highlights the limitation of using a single read-out (e.g., NF-κB signalling) to indicate the overall efficacy of an adjuvant. Mechanistically, the trimeric conjugate (**37**) was found to activate both TRIF and MyD88 signalling pathways, with contributions by the TLR4 (indole-mediated) and TLR9 (CpG-mediated) pathways being particularly important. When administered as an adjuvant in a model vaccination system using an inactivated vaccinia virus, the trimer (**37**) led to an increase in the number of reactive antigens compared to the unlinked mixture of PAMPs. It was suggested that this might be due to differences in the order in which the agonists activate the TLRs when they are conjugated versus unconjugated [19], again demonstrating how conjugation can lead to synergistic immune cell activation.

Following their seminal work [19], Esser-Khan’s group then made five new trimeric PAMPs: TLR4-TLR7-TLR9 (**38**), TLR2/1-TLR4-TLR7/8 (**39**), TLR2/1-TLR4-TLR9 (**40**), TLR2/6-TLR4-TLR7/8 (**41**), and TLR2/6-TLR4-TLR9 (**42**) [86]. These conjugates used the same core as that in the original work but included PEG12 linkers on the TLR4 and TLR7 agonists (Figure 5) [86]. Of these, the TLR2/1 and TLR2/6 agonists contained a Pam_3_CSK_4_ (**4**) or Pam_2_CSK_4_ (**2**) linked to a fluorescein isothiocyanate (FITC) fluorophore, which was used for quantification and identification. In vitro, the TLR2-TLR4-TLR7 trimers **39** and **41** led to reduced levels of Mφ-mediated NF-κB signalling compared to the unconjugated PAMPs, while no difference was seen for the other trimers **38**, **40** and **42**. Similar results were observed when the trimers were assessed for their ability to upregulate interferon stimulatory genes (ISGs) in Mφ. The cytokine production by BMDMs revealed a shift towards a more T_H_1 profile for the Pam_3_CSK_4__indole_CpG (**40**) and indole_Lox_CpG (**38**) trimers and a T_H_2 type response for the Pam_2_CSK_4__indole_quinoline (**41**) and Pam_2_CSK_4__indole_CpG (**42**) trimers, although the Pam_3_CSK_4__indole_quinoline (**39**) conjugate produced relatively low levels of all cytokines compared to the other trimers and admixed PAMPs.

In general, the increase in vitro cytokine production for the trimer versus the individual PAMPs was greatest for Pam_3_CSK_4__indole_CpG (**40**) [TLR2/1_4_9] and indole_Lox_CpG (**38**) [TLR4_7_9]. In vivo, differences were observed in the level of systemic cytokines following the administration of the conjugate and admixed PAMPs and between the different trimers. However, for the most part, the trimers elicited no to modest cytokine production, with the cytokine response being greatest for those conjugates containing the TLR9 agonist CpG; most notably, Pam_3_CSK_4__indole_CpG (**40**). This observation is in agreement with the in vitro data. In addition, weight loss often, but not always, correlated with cytokine production, with rapid weight loss being associated with a cytokine storm in response to the Pam_2_CSK_4_ + indole + CpG admix combination. However, there were some anomalies. For example, there was no weight loss associated with the administration of Pam_3_CSK_4__indole_CpG (**40**) despite this conjugate leading to modest cytokine production in vivo.

The trimers were then assessed for their potential as an adjuvant in subunit vaccinations for Q-Fever, which is a zoonotic disease caused by the pathogen *Coxiella burnetii* antigen. Using conjugated PAMPs as adjuvants helped improve the immune response to *C. burnetii* antigens in many circumstances [87]. For example, when combined with the antigen CBU-1910, Pam_2_CSK_4__indole_quinoline (**41**) led to greater antibody titres and less weight loss compared to the admixed PAMPs, although it did not confer protection. Of all trimers, only indole_lox_CpG (**38**) [TLR4_7_9] led to a significant increase in serum IFN-γ, despite only modest antibody titres. Indole_lox_CpG (**38**) and Pam_3_CSK_4__indole_CpG (**40**) [TLR2/1_4_9], which also showed a T_H_1-bias, were then further investigated as adjuvants when using a broader selection of *C. burnetii* antigens (MHC class I epitopes) and the existing nanoemulsion adjuvant AddaVax. Both trimeric PAMP-containing vaccines led to increased IgG2c antibodies (T_H_1-skewing) and conferred partial protection against a live *C. burnetii* challenge. For the TLR4_7_9 conjugate (**38**), there was also no significant difference in weight loss or fever compared to the PBS control. Taken together, this data illustrates the potential of these two trimeric conjugates as vaccine adjuvants, although neither was as protective as the commercial vaccine Q-Vax [87].

## 3. NOD2-TLR Agonist Conjugates as Vaccine Adjuvants

### 3.1. NOD2 Signalling Pathways

**Figure 6:**
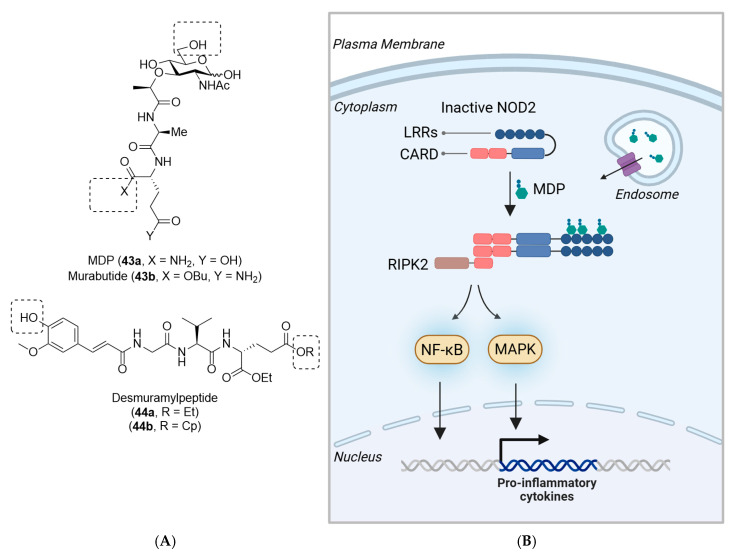
(**A**) Structures of NOD2 ligands used in conjugate syntheses. Sites useful for conjugation have been highlighted using dotted boxes. (**B**) NOD2 signalling pathway. NOD2 exists in an inactive form in the cytosol, where MDP can be released after endocytosis and released from the endosome. Upon binding of MDP to the leucine-rich repeats (LRRs) of NOD2, a conformational change is induced that releases CARD domains for oligomerisation. Oligomerisation of NOD2 allows interaction with RIPK2 kinase via CARD domains, which activates NF-κB and MAPK signalling pathways for the production of pro-inflammatory cytokines. NOD2 is also known to respond to ssDNA and, in conjunction with MAVS, activate IRF3 for the production of type I IFNs.

NOD2 is an intracellular PRR that is activated by muramyl dipeptide (MDP, **43a**, Figure 6A) from bacterial cell walls [88,89]. Upon activation, NOD2 oligomerises to bring its carbohydrate recognition domains (CARDs) into proximity, which ultimately leads to NF-κB activation and the expression of pro-inflammatory cytokines (Figure 6B) [89]. NOD2 is also known to respond to ssDNA and, in conjunction with the mitochondrial antiviral signalling (MAVS) adaptor protein, activates interferon regulatory factor-3 (IRF-3) to produce type I IFNs [90]. Previous work has shown that codelivery of NOD2 and TLR agonists can synergistically enhance cytokine production by hPBMCs, DCs and mouse Mφ [91,92,93]. However, care must be taken when using MDP in vivo as it has significant toxicity, most notably, pyrogenicity (the induction of fever) [24]. This can be mitigated via careful conjugate design.

### 3.2. NOD2 Ligands Used in PAMP Conjugates

In 2016, the crystal structure of adenosine diphosphate (ADP)-bound NOD2 was published, revealing the inactive conformation of NOD2 [88]. While the crystal structure of ligand-bound NOD2 is yet to be determined, it is known that the leucine-rich repeat (LRR) domain of NOD2 can bind MDP (**43a**) (Figure 6A) with high affinity and that both the carbohydrate and peptide portions of MDP are required for binding [94]. The use of a surface plasmon resonance assay indicated that NOD2 recognition of MDP requires binding to the free anomeric centre and amide 2-position of the carbohydrate and to the peptide terminal amide of d-isoglutamine [95]. Notwithstanding, the development of NOD2-chimeras necessitates chemical conjugation to the NOD2 ligand, which, in the case of MDP, has sometimes resulted in linkages at the anomeric position, as such adducts are chemically more accessible.

Many modifications have been made to MDP to determine the SAR around this class of NOD2 agonist [24]. Notably, murabutide (**43b**), which contains d-glutamine (rather than d-isoglutamine) and has an *n*-butyl ester at the *C*-terminus of the dipeptide, is immunomodulatory but not pyrogenic [96] and is often used in research. The conjugation of lipophilic groups to MDP (e.g., MDP–cholesterol or MDP–octadecane) can also reduce toxicity, although it is thought that these need to be hydrolysed in the cell to be effective NOD2 ligands [97]. More recently, the use of *N,O*-hydroxylamine linkers provided synthetic MDP derivatives that could bind to NOD2 and signal through NF-κB [98], although again, it remains to be conclusively determined whether hydrolysis of this linker occurs inside the cytosol before NOD2 activation in vivo [99]. Within the context of PAMP conjugates, the ability to “mask” the toxicity of MDP via the incorporation of a hydrolysable or non-hydrolysable linker has meant that, despite its toxicity, MDP (**43a**) has been successfully used in the design of several PAMP conjugates.

### 3.3. NOD2-TLR Agonist Conjugates

#### 3.3.1. Summary of NOD-TLR Agonist Conjugates Prepared to Date

There is much evidence that the coadministration of NOD2 and TLR agonists leads to synergistic immune cell activation [91,92,93]. This has led to the synthesis of conjugates containing agonists for both PRRs, including the NOD2 ligands MDP (**43a**), murabutide (**43b**), and desmuramylpeptides (**44a** and **44b**) [79,100], and ligands or TLR2 [Pam_2_Cys (**1**) or Pam_3_CSK_4_ (**4**)], TLR4 [RC529 (**7**), AZ617 (**9**)] or TLR7 [CL264 (**11a**)] (Table 3) [53,79,101,102,103,104,105].

#### 3.3.2. NOD2-TLR2 Agonist Conjugates

**Figure 7:**
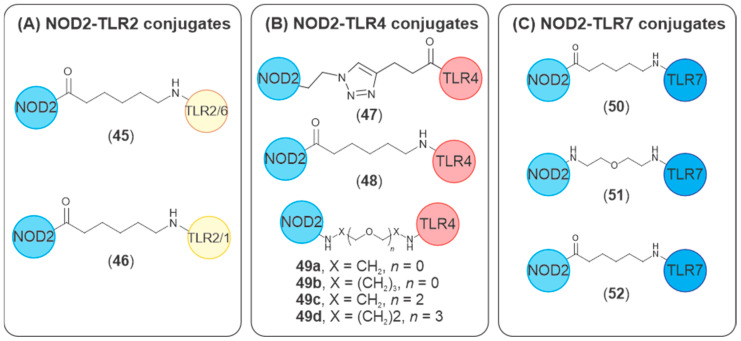
Schematic representations of NOD2 agonist conjugates with (**A**) TLR2, (**B**) TLR4, and (**C**) TLR7 agonists.

The first of the NOD2-TLR agonist conjugates was synthesised in 2014 by Pavot et al. [53]. The conjugate CL429 (**45**) contained a benzyl analogue of murabutide (**43b**) as the NOD2 agonist and Pam_2_Cys (**1**) as the TLR2 agonist, with the two being linked via a short spacer (Figure 7A). The conjugate activated both NOD2 and TLR2, as evidenced using NOD2 or TLR2 HEK reporter cells, and induced a synergistic increase in DC maturation markers (CD83, CD80, CD86 and MHCII) compared to the administration of murabutide + Pam_2_Cys. A synergistic increase in IL-1β, TNF, IFN-γ, IL-6 and IL-12p70 by DC was also observed following treatment with the conjugate. Notably, the admixed ligands did not lead to IL-12p70 production. The induction of systemic and mucosal immune responses after parenteral immunisation in mice using nanoparticle vaccines containing the Gag p24 (HIV-1) antigen and the conjugate (compared to the individual PAMPs and admixed PAMPs) was then explored. The conjugate (**45**) significantly increased IgG and IgA titres up to 2 logs in comparison with all other formulations, with the predominant IgG subclass being IgG1 (T_H_2) for all groups. In addition, the conjugate and admixed ligands also led to an increase in IgG2a, thus resulting in a more balanced T_H_1:T_H_2 ratio. The authors reported no apparent toxicity associated with the chimera and that it was a potent inducer of immune effector cell chemotaxis. Taken together, this data represented the promising potential of the conjugation of NOD2 ligands to another PAMP, with the conjugate later being called CL429 (which is now commercially available). Shortly thereafter, CL429 (**45**) was found to suppress virus-induced inflammation in vivo and prevent the lethal sequelae of acute respiratory virus infection [102]. This response to the conjugate was greater than that to the combined individual PAMPs and was found to be dependent on both NOD2 and TLR2 signalling.

More recently, a series of PAMP chimeras was prepared and tested for their ability to act as adjuvants in vaccines for infectious diseases, as well as within the context of cancer immunotherapy (to be peer-reviewed) [79]. As part of these studies, the NOD2_TLR2/1 chimera (**46**) was synthesised, which contained the desmuramylpeptide NOD2 agonist **44a**, which was previously identified by the same group [100], and the small-molecule TLR2/1 agonist CU-T12-9 (**6**), which were linked through a 6-amino-hexanoic acid linker. This PAMP combination led to substantial BMDC IL-6 secretion, and gene expression analysis revealed that conjugate (**46**) enriched the “IL-17 signalling pathway”.

#### 3.3.3. NOD2-TLR4 Agonist Conjugates

Three TLR4-NOD2 ligand conjugates have been developed that contain different PAMPs but also a variety of hydrolysable or more metabolically stable linkers [103,104]. In 2024, conjugate **47** was prepared, which incorporates MDP (**43a**) as the NOD2 agonist and RC529 (**7**) as the TLR4 agonist (Figure 7B) [103]. Here, Ding et al. synthesised RC529 (**7**) with an alkyne handle at the 6-position of the carbohydrate and, using a Cu(I)-catalysed click reaction, conjugated this to an MDP derivative that contained an azide linked to its anomeric position via an ethyl chain. The immunomodulatory properties of RC529-MDP (**47**) were then explored following the injection of mice with liposomal formulations of the various PAMP combinations. The analysis of serum cytokine levels revealed that RC529-MDP (**47**) led to a 17-fold increase in IL-6 compared to a 1.04-fold increase by MDP + RC529. To learn more about the signalling pathways, BMDMs were then treated with NOD2 or TLR4 inhibitors in vitro, and IL-6 production was measured. The conjugate activated both signalling pathways; however, the inhibition of TLR4 signalling had a major effect on IL-6 secretion, thus suggesting that TLR4 mediates the internalisation of RC529-MDP (**47**) to facilitate subsequent NOD2 signalling. The conjugate was also shown to lead to significantly more APC maturation compared to the admixed PAMPs (as evidenced by CD80 and CD86 upregulation on BMDMs). Given these promising results, the adjuvanticity of RC529-MDP (**47**) was explored using OVA as a model antigen. Total OVA-specific IgG titres were significantly greater for RC529-MDP (**47**) (compared to admixed PAMPs) and were predominantly IgG1, indicating a T_H_2-bias. In recall experiments, the conjugate (**47**) led to a significant increase in TNFα and IFN-γ secretion by both CD4+ and/or CD8+ T cells compared to RC529 + MDP. The conjugate (**47**) also led to a significantly higher proportion of memory CD4+ T cells and CD8+ T cells compared to treatment with OVA alone, with the CD8+ T cell response to the chimera being significantly greater than that to the admixed PAMPs. The conjugate (**47**) did not have any discernible toxicity, as determined through the histological evaluation of various tissues and the measurement of alanine aminotransferase and aspartate aminotransferase in the serum. Taken together, this data demonstrates the significant advantages that can be gained by conjugating NOD2 and TLR4 agonists. In this example, there was evidence to suggest that NOD2 signalling is augmented via TLR4-mediated endocytosis.

The effect of linker chemistry on the immunomodulatory activity of two NOD2_TLR4 chimeras was recently assessed by Paradiso et al. [104]. This followed earlier work in which the group previously made an NOD2_TLR4 chimera (**48**) containing a pyrimido-indole derivative (**8c**) and an in-house NOD2 agonist (**44b**), which lacked immunostimulatory activity [79]. For their new studies, Paradiso et al. developed two new NOD2_TLR4 chimeras (**49**): a first series containing the same ligands [pyrimido-indole (**8c**) and NOD2 agonist (**44b**)] but with a variety of different linkers (e.g., with alkyl or PEG chains of different length) and a second series containing the NOD2 ligand (**44b**) and AZ617 (**9**) [106], again using alkyl or PEG linkers of different lengths. The solubility of the different chimeras was also assessed. In general, those conjugates containing the bulkier TLR4 AZ617 (**9**) had reduced solubility compared to their precursor PAMPs, while the conjugates containing the pyrimido-indole (**8c**) had solubility more akin to the individual PAMPs (with the exception of the chimera with the piperazine linker). The inclusion of the PEG linker also improved solubility. The ability of the chimeras to activate NOD2 or TLR4 HEK293 reporter cells, as measured by NF-κB activation, was then explored. Those chimeras containing AZ617 (**9**) generally retained their ability to signal through NOD2, while compounds with the indole (**8c**) led to a loss of activity. Signalling through TLR4 was poor for all agonists compared to that of the parent compounds; however, once again, those derivatives containing AZ617 (**49**) exhibited better TLR4 signalling compared to their indole counterparts, particularly the least soluble derivative (containing the piperazine linker). The ability of the conjugates to lead to cytokine production by hPMBCs was also modest, with the piperidine-linked AZ617-NOD2 ligand conjugate (**49**) once again showing the best response (IL-6 production) [albeit, the immune response was less than that exhibited by the admixed PAMPs]. Taken together, linking the NOD2 and TLR4 agonists led to decreased activity compared to the coadministered PAMPs for this conjugate. This was mainly attributed to an inability of the compound to signal via TLR4.

#### 3.3.4. NOD2-TLR7 Agonist Conjugates

The linkage of TLR7 agonist CL239 (**11c**) via an amide on the benzyl group and an ester at the 6-position of MDP (**43a**) led to the formation of the TLR7-NOD2 chimera (**50**) [105] (Figure 7C), which was found to have exceptional adjuvant properties. The TLR7-NOD2 chimera (**50**) existed as stable particles, with an average particle size of 300 nm +/− 70 nm, which the authors believed would favour multi-affinity presentation and uptake by APCs. In vitro, the chimera (**50**) activated both NOD2 and TLR7 to a greater extent than CL239 (**11c**) alone, leading to human DC maturation (e.g., CD40, CD80, CD86 and MHC-II upregulation), a synergistic increase in cytokine secretion (IFN-γ, IL-10, IL-6, IL-1β and IL-8) compared to that elicited by the individual PAMPs, and a substantial increase in memory CD8+ T cells. Notably, the chimera (**50**) elicited autophagy in human cells, which is positively associated with cytokine production and the survival of memory B cells. The TLR7_NOD2 chimera (**50**) was also able to induce protective mucosal immune responses against the HIV-1 p24 antigen without any associated toxicity, and it induced higher IgG titres compared to all other groups, with IgG2a being the predominant subclass. A significant increase in systemic and mucosal IgA was also observed in response to the TLR7_NOD2 chimera (**50**), while no IgA was observed in response to the other adjuvants. The stimulation of splenocytes or lung CD4+ and CD8+ lymphocytes with antigens induced a strong IFN-γ and IL-17 response for the group containing the conjugate, which is suggestive of a T_H_1/T_H_17 immune response. Finally, the chimera was able to induce significant protection from viral vaccinia challenge when using the NP-p24 antigen and a high reduction in viral titres in the lung, thus demonstrating its potential as a versatile adjuvant for prophylactic and therapeutic vaccines.

Guzelj et al. also synthesised two NOD2_TLR7 chimeras containing their in-house desmuramylpeptide NOD2 agonist (**44b**) and the purine-based TLR7 agonist CL239 (**11c**) [101]. The first chimera (**51**) contained a 6-aminohexanoic acid spacer connected to the phenol of NOD2 via a cleavable ester, while the second chimera (**52**) incorporated a bis(2-aminoethyl)-ether spacer attached to the ω-carboxylic acid of the d-glutamic acid moiety through a more metabolically stable amide bond. Both chimeras showed improved TLR7 signalling compared to the purine-based TLR7 agonist (**12**), with this improvement being most notable for chimera **51**. However, only chimera **52** activated NOD2 with similar potency to MDP, while chimera **52** exhibited poor NOD2 signalling. Both chimeras elicited extensive pro-inflammatory responses by hPBMCs, as evidenced by significant increases in IL-1β, IL-6, IL-8, IL-10, and TNF production, which were greater than those observed in response to the admixed PAMPs for all cytokines except IL-8. Chimera **52** exhibited the stronger response, with further support for this being obtained from transcriptome analysis. Using a mixed-lymphocyte assay, both chimeras induced the antigen-specific activation and proliferation of CD4^+^ and CD8^+^ T cells and led to significant and similar levels of IL-6, IL-17A, TNF, and IFN-γ to those induced by the admixed PAMPs. Using OVA as a model antigen, the adjuvanticity of the NOD2_TLR7 chimera (**52**) (but not **51**) was investigated and found to lead to significant total IgG, IgG1 and IgG2a compared to OVA + MDP or OVA + TLR7 agonist CL239 (**12**). No direct comparison was made to the coadministration of MDP (**43a**) + CL239 (**11c**).

## 4. Other PAMP-PAMP Conjugates (Containing RIG-I, Mincle, STING)

### 4.1. Summary of RIG-I, Mincle and Sting Agonist PAMP Conjugates

**Figure 8:**
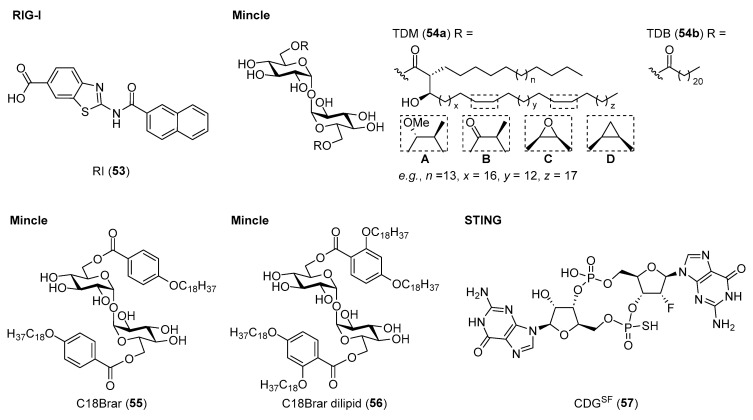
RIG-I, Mincle and STING ligands used in PAMP chimeras.

In addition to the development of PAMP-PAMP conjugates containing TLR or NOD2 ligands, studies have been extended to PAMP conjugates containing ligands for Retinoic acid-inducible gene (RIG-I) [77], Macrophage-inducible C-type lectin receptor (Mincle) [99,107] and STING [108] [Table 4]. Thus far, only a small selection of ligands have been used for these conjugates, including RI (53) for RIG-I signalling [109]; trehalose dimycolate (54a) [110], C18Brar (55) [111,112,113], and C18Brar dilipid (56) [99] for Mincle signalling; and cyclic dinucleotides (CDNs, 57) for signalling via STING [108] (Figure 8).

### 4.2. PAMP Chimeras Containing Ligands for Retinoic Acid Signalling Pathways

#### 4.2.1. Retinoic Acid Signalling Pathways

**Figure 9:**
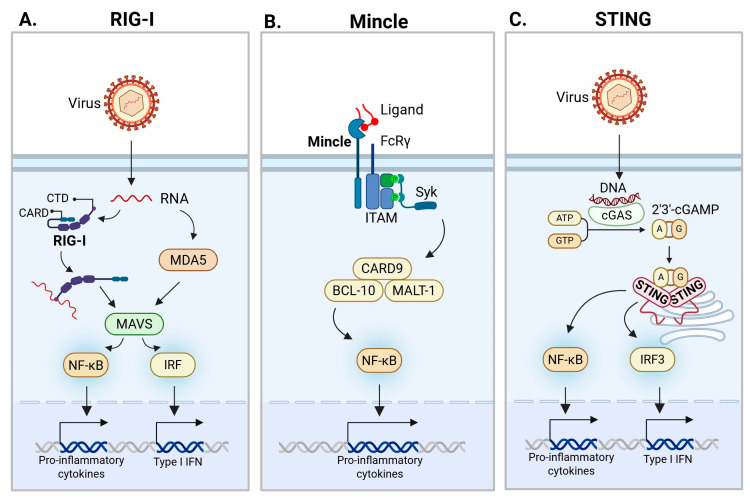
(**A**) RIG-I signalling pathways. Viral RNA binding to inactive RIG-I via the CTD leads to a conformational change releasing CARD domains for oligomerisation with MDA5. The RIG-I-MDA5 oligomer interacts with the CARD domain of MAVS, leading to the recruitment of NF-κB and IRF transcription factors that promote transcription of cytokines. (**B**) Mincle signalling pathways. Activation of Mincle by ligand binding leads to coupling with adaptor molecule FcRγ, phosphorylation of the ITAM motif, and recruitment of Syk family kinases. Subsequent formation of the Card9-Bcl10-Malt1 signalling complex leads to activation of NF-κB and transcription of pro-inflammatory cytokines. (**C**) STING signalling pathways. Binding of viral DNA to cGAS facilitates the production of 2′3′-cGAMP, which binds to STING, leading to translocation of the complex from the ER to the Golgi, where it can activate NF-κB and IRF transcription factors that promote transcription of cytokines.

Retinoic acid-inducible gene-I-like receptors (RLRs) are intracellular cytoplasmic PRRs that recognise viral RNA produced during infection [6,114]. RIG-I is the most well-characterised RLR and binds defined molecular features in viral RNA that are generally not present in host RNA, including uncapped 5′-diphosphate or triphosphate groups [114]. In uninfected cells, RIG-I exists in an inactive conformation. When it binds its ligand, the C-terminal domain (CTD) changes shape, exposing the two amino-terminal caspase activation and recruitment domains (CARDs), which then oligomerise with MDA5. The resulting RIG-I–MDA5 complex interacts with the CARD domain of mitochondrial antiviral-signalling protein (MAVS), triggering recruitment of the NF-κB and IRF transcription factors. This initiates downstream signalling and promotes type I interferon (IFN) production [Figure 9A]. RIG-I signalling can also lead to programmed cell death. Several small molecules have been identified that can induce the conformational change necessary for RIG-I signalling [109,115], although the only RIG-I ligand that has been used in PAMP conjugates to date is RI (**53**) [109] (Figure 8).

#### 4.2.2. PAMP Chimeras Containing Ligands for RIG-I

**Figure 10:**
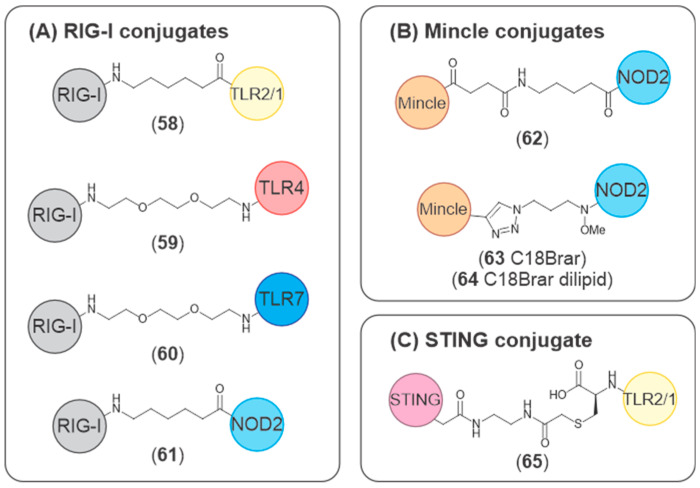
(**A**) RIG-I ligand conjugates, (**B**) Mincle ligand conjugates, and (**C**) STING ligand conjugates.

There is evidence to suggest that synergistic immune responses can be observed via the activation of RIG-I pathways and TLRs [116], although it has been postulated that RIG-I inhibits NOD2-induced NF-κB activation [117]. To explore these phenomena further, RIG-I ligand-PAMP conjugates are the latest addition in the development of new classes of PAMP conjugates [79] (still to be peer-reviewed). Here, the authors used the ligand RI (**53**), an analogue of their in-house RIG-I ligand KIN1148 [109] that is equipped with an ester handle for conjugation, to prepare four RIG-I-targeting PAMP-PAMP conjugates: RIG-I-TLR2/1 (**58**), RIG-I-TLR4 (**59**), RIG-I-TLR7 (**60**) and RIG-I-NOD2 (**61**) [79] (Figure 10A). In the initial screen, cytokine production from hPBMCs in response to the conjugates was measured and compared to the response elicited using the relevant admixed ligands. The coadministration of RIG-I and TLR2/1 ligands led to modest TNF, IL-6 and MCP-1 production; however, conjugation of these PAMPs (conjugate **58**) significantly reduced this activity. Neither coadministration nor the PAMP conjugates of RIG-I ligands with TLR4 or NOD2 ligands (conjugates **59** and **61**, respectively) induced cytokine production. Conjugation of RIG-I and TLR7 ligands (conjugate **60**) significantly enhanced cytokine production compared to coadministration of these ligands. From this initial screen, the TLR7-RIG-I agonist conjugate (**60**) was tested in vivo alongside the TLR4-TLR7 agonist conjugate (**33**) (which was also prepared by the same group). The TLR7-RIG-I ligand conjugate (**60**) reduced tumour volume in a murine B16F10 tumour model; however, the tumour-suppressive activity of this compound was not as pronounced as the response to the TLR4-TLR7 agonist conjugate (see Section 2.4.4).

### 4.3. PAMP Chimeras Containing Ligands for Mincle

#### 4.3.1. Mincle Signalling

Mincle is found on the cell membranes of cells in the myeloid lineage [118] and has been a recent target for the development of vaccine adjuvants [119]. Upon ligand binding, Mincle couples with the Fc receptor gamma (FcRγ) signalling chain that contains an immunoreceptor tyrosine-based activation motif (ITAM), which then leads to the activation of the kinases Syk and Erk and the formation of the CARD9/Bc110/Malt1 complex [Figure 9B] [110,119,120]. This then leads to the activation of NF-κB signalling and the expression of various gene products, which are predominantly inflammatory, and a T_H_ immune response that is typically T_H_1/T_H_17 in phenotype [119]. Mincle recognises a variety of PAMPs or analogues thereof, although, within the context of Mincle-PAMP conjugates, these have been limited to trehalose-6,6′-dimycolate (TDM, **54a**) [104] and C18Brartemicin derivatives (**55**) and (**56**) (Figure 8) [111,112,113].

#### 4.3.2. Mincle-NOD2 Conjugate Design

To date, three Mincle-PAMP conjugates have been synthesised [93,107], both of which include MDP (**43a**) as the ligand for NOD2, conjugated via either a non-cleavable [107] or cleavable [99] linker to the terminus of the lipophilic chains on a trehalose glycolipid (Figure 10B). The coadministration of the Mincle ligand trehalose dibehenate (TDB, **43b**) and either NOD (c12-*iE*-DAP) or NOD2 (L18-MDP) ligands can synergistically enhance pro-inflammatory responses via the coactivation of NF-κB and AP-1 transcription factors [20,121], thus supporting the choice of developing chimeric Mincle-NOD2 agonists.

Like other *C*-type lectins, Mincle contains a sugar-binding EPN (Glu-Pro-Asn) motif and a Ca^2+^ cofactor, which are essential for binding to the *trans*-diequatorial 3-OH and 4-OH positions of a glucose moiety in trehalose [122,123,124,125]. Another binding site accommodates the second glucose monomer. Next to the sugar-binding sites are major and minor hydrophobic grooves that accommodate the lipophilic chains. These studies, as well as others involving molecular modelling [111], and the formation of a functionalised α,α′-trehalose glycolipid containing a fluorescent reporter group at one of the terminal lipids [126], suggest that the development of Mincle agonist–PAMP conjugates that are linked at the terminus of a suitably long (e.g., >C12) carbon chain should avoid negative interactions with the Mincle binding site and retain their Mincle-mediated signalling capacity. Indeed, the TDM-MDP derivative (**62**) [107] and the C18Brar-NOD2 derivatives (**63**) and (**64**) [99] meet these design features, although the TDM-MDP chimera (**62**) was synthesised before Mincle was identified.

The TDM-MDP conjugate (**62**) contained a succinic acid linker that was conjugated to MDP at the 6-position of the carbohydrate [107]. Given that little was known about MDP binding to NOD2 at the time, this design nonetheless left the anomeric and amide 2-positions of the carbohydrate free, as well as the peptide terminal amide of d-isoglutamine, which are all deemed important for NOD2 activation [95]. The TDM-MDP chimera (**62**) activated murine peritoneal Mφ and led to delayed-type hypersensitivity responses in guinea pigs to levels similar to those induced by MDP alone [107]. Further studies were not undertaken with this chimera.

In later work, Dangerfield et al. developed two Mincle-NOD2 chimeras that were designed to release MDP in the lysosome [99]. Here, the anomeric position of MDP was conjugated to a Mincle ligand [either C18Brar (**63**) or C18Brar dilipid (**64**)] using a pH-sensitive cleavable linker. It is known that the toxicity of MDP can be mitigated via the inclusion of a lipid chain [127], so it was anticipated that the chimeras would have reduced toxicity compared to MDP alone. In vitro, enhanced IL-6 and IL-23 cytokine production was observed in response to the MDP-C18Brar dilipid conjugate compared to coadministration of these ligands. Using an in vivo vaccine model with OVA as the model antigen, the MDP-C18Brar dilipid (**64**) had reduced toxicity and led to an enhanced memory recall response compared to the coadministration of the two ligands. The MDP-C18Brar conjugate (**63**) led to similar recall responses compared to the coadministered PAMPs. However, conjugate **63** had enhanced antibody production compared to the coadministration of MDP (**43a**) and C18Brar (**55**). Moreover, both conjugates were used at very low doses (0.04 μmol/dose). Taken together, this data supports the potential of conjugating Mincle ligands with NOD2 ligands to augment vaccine efficacy while also allowing for adjuvant sparing. The data also highlights the effect that subtly different ligands can have on the ensuing immune response.

### 4.4. Chimeras Containing Ligands for STING

#### 4.4.1. STING Signalling

The PRR STING is located on the cytosolic face of the endoplasmic reticulum (ER) and is an indirect sensor for aberrant cytosolic DNA [128]. STING works in tandem with cyclic GMP-AMP synthase (cGAS), which itself recognises aberrant double-stranded DNA in the cytosol and consequently synthesises 2′3′ cyclic GMP-AMP (cGAMP) [Figure 9C]. cGAMP then acts as a secondary messenger, activating STING and leading to the translocation of the complex from the endoplasmic reticulum (ER) to the Golgi, where it can activate NF-κB and IRF transcription factors that promote production of type I INFs [129]. STING activation can also lead to a range of other downstream events, including NF-κB activation, cell death, translation inhibition, and metabolic reprogramming [128]. In addition to cGAMP, STING can bind to a range of other cyclic dinucleotides (CDNs, **57**), which also induce the production of type I interferon and inflammatory cytokines, and which have shown great potential for use in cancer immunotherapy [130]. STING agonists have synergistically enhanced immune responses in combination with TLR ligands, including those for TLR7 [131] and TLR9 [132], particularly within the context of cancer immunotherapy [131,132,133]. Perhaps it is therefore not so surprising that a STING (CDN-containing)-TLR2/1 agonist conjugate has been developed and tested for its ability to lead to a reduction in tumour burden in a B16-OVA murine model of cancer [108].

#### 4.4.2. PAMP Conjugates Containing STING Agonists

Hu et al. synthesised a Pam_3_CSK_4_-CDG^SF^ conjugate (**65**) to activate both the STING and TLR2/1 pathways (Figure 10C) [108]. No significant difference in cytokine production by RAW 264.7 Mφ was seen in response to the conjugate compared to the admixed PAMPs in vitro. In contrast, in vivo, the conjugate strongly inhibited tumour growth compared to coadministration of the PAMPs in a B16-OVA tumour model. In this model, the conjugate enhanced cellular responses through the production of IgG antibodies and boosted the secretion of T_H_1 inflammatory cytokines, leading to antigen-specific cytotoxic T lymphocytes and enhanced tumour immunity.

## 5. Conjugated PAMP Chimeras: Where to from Here?

While a variety of PAMP-PAMP conjugates have been synthesised, predicting the functional outcomes of PRR combinations remains challenging, particularly in vivo, where responses are strongly influenced by the selected antigen and vaccine formulation [5]. For admixed PAMP combinations, computational approaches are emerging to predict combinatorial effects, with one study suggesting that the in vivo effect of multi-adjuvant combinations may be predicted through the systematic characterisation of single and pairwise combinations [134]. However, these predictions may not capture the effect of covalent conjugation, which can change ligand efficacy, pharmacokinetic profiles, and cell targeting compared to the unconjugated form [78,135,136].

From the studies reported herein, it is clear that effort has been made to correlate the effect of PAMP conjugation on the immune response, and it has been suggested that the PAMP conjugate will favour the immune profile of the most immunostimulatory PAMP. For example, conjugates containing CpG or Pam_3_CSK_4_ typically led to a T_H_1 bias, while the inclusion of the TLR2/6 agonist Pam_2_CSK_4_ can favour a T_H_2 response [86,87]. However, it is not always easy to predict which combination of PAMPs will be efficacious and in what context. More work is required in this regard. For example, several TLR2_TLR7 PAMP conjugates appear to have excellent adjuvanticity across a number of experimental set ups, including when using HIV antigens [Pam_2_Cys_CL307 (**19**)] [80], in allergy models [Pam_2_CSK_4__CL307 (**20**)] [81], and in antitumour models [Pam_2_CSK_4__imidazoquinoline (**23**)] [82], while other TLR2_TLR7 ligand conjugates appear to be less effective than the admixed PAMPs [78]. There has also been some suggestion that the less potent PAMPs have less effect on the ensuing immune response, which may indeed be true, although the incorporation of such ligands into PAMP trimers nonetheless influenced gene transcription [86,87]. It has also been suggested that covalently linking CpG to another large PAMP has a more deleterious effect on TLR9 signalling compared to conjugation to a lower-molecular-weight PAMP [72,84]. Whether this is a general feature that applies to the conjugation of other high-molecular-weight PAMPs remains to be seen.

**Figure 11:**
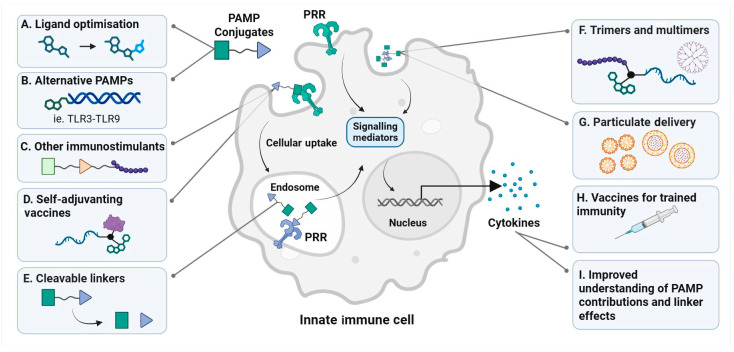
Areas for future development. (**A**) Optimisation of ligands for PRRs. (**B**) Alternative PAMPs for conjugation, including those for PRRs not previously explored. (**C**) Combination with other immune-stimulating molecules, such as inflammasome-activating peptides. (**D**) Conjugation to antigens for self-adjuvanting vaccines. (**E**) Exploration of hydrolysable linkers for endosomal targeting. (**F**) Development of higher-order PAMP conjugates. (**G**) Delivery of conjugates as part of particulate/polymer carriers. (**H**) Use of conjugates for vaccines for trained immunity. (**I**) Improved understanding of PAMP contributions and linker effects.

In addition to PAMP structure, in vitro versus in vivo immune responses also need to be considered. For example, singular in vitro readouts are not sufficient for a detailed picture of the immunomodulatory potential of a conjugate. This was noted for several TLR conjugates that led to very little change in NF-κB signalling compared to the admixed PAMPs but resulted in an increase in cytokine responses by BMDCs [19]. In addition, several PAMP conjugates containing NOD2 ligands resulted in very little difference in cytokine production by Mφ or DC compared to the admixed PAMPs in vitro, while the same conjugates led to very promising in vivo adjuvant activity [99,108]. Taken together, this points to the need for a greater number of studies to better understand how the choice and combination of PAMPs affects both in vitro and in vivo immune responses (Figure 11).

Within the context of NOD2 ligands, many seem to be well tolerated in conjugate design and give PAMP conjugates that can lead to immune cell synergy (compared to admixed PAMPs) [53,80,99,102,103]. However, once again, more studies are required to tease apart the differences in immunomodulatory profiles generated by each class of conjugate. Studies into the preparation and evaluation of RIG-I, Mincle, and STING agonist conjugates are in their infancy. Promising results have been obtained for the potential of Mincle-NOD2 ligand conjugates as vaccine adjuvants (with antigen sparing and reduced toxicity) [99] and for STING-TLR2/1 ligand conjugates for anticancer immunotherapy [108]; however, compared to studies using TLR ligands, targeting these PRRs is relatively unexplored. Similarly, only one group has thus far constructed RIG-I-agonist-containing PAMP conjugates [77]. This is clearly an area that could be further explored, as is the development of PAMP conjugates containing TLR3, TLR5 and/or TLR9 agonists.

Concerning the choice of PAMP for chimeric PAMP conjugates, further work is required to determine if the conjugation of TLR ligands for extracellular PRRs alone can lead to immune cell synergy. This is seen with the TLR2-TLR4 conjugates, where, to date, the admixed TLR2/TLR4 ligands have led to greater immune cell responses than the chimeras. We tentatively speculate that this might be due to the challenges associated with optimally targeting extracellular TLRs that need to aggregate to lead to an optimal immune response (see Section 2.4.2). Similarly, the trimeric TLR2-TLR4-TLRX conjugates do not fare any better than the coadministration of the three individual PAMPs, although there can be subtle differences in immune response, e.g., reduced weight loss for the Pam_2_CSK_4_–pyrimido-indole–imisazoquinoline (TLR2-TLR4-TLR7/8) conjugate **41** compared to the individual PAMPs.

The studies herein also suggest that various ligands are well tolerated in PAMP conjugate design, although immune cell activation is influenced by linker length and the site of conjugation, and such effects can be difficult to predict. While much is known about the preferred binding modes for the individual PAMPs (as discussed in Section 2.2, Section 2.4.2 and Section 3.2), the ease of synthesis sometimes means that conjugation may not be at the optimal site and PRR signalling can be reduced. However, reduced PRR binding/signalling abilities can sometimes be offset by increases in the synergistic effects of combining two or more ligands, making this a double-edged sword [84]. In other instances, conjugation of the agonists can lead to vastly reduced immunostimulatory activity. This was seen in studies by Jakopin and co-workers, who determined that imidazoquinolines do not tolerate the side chain amine or aminoquinoline positions as conjugation sites without significant loss in activity [79], and in studies by Kimani et al., where several conjugates [e.g., Pam_3_CSK_4_–indole conjugate **17** (TLR2/1-TLR4)] led to poor immunostimulatory activity in vitro [78], which could be attributed to a disruption in the receptor–agonist interactions. Similarly, the length and type of linker also influence the immune response. Esser-Kahn and co-workers studied PAMP conjugates with varying linker lengths [72,78] and found that spatially constrained TLR agonists limited their ability to activate individual TLRs [84]. However, it was not possible to predict what lipid length would lead to optimal immune responses, as this depended on the PAMPs that were conjugated. Such limitations may point to the need for alternative PAMP conjugates or, where relevant, the use of cleavable linkers, of which there are many for the intracellular release of ligands [137,138], such as those for TLR3, TLR7, TLR8 and TLR9, as well as NOD2.

In addition to potentially circumventing any negative effects on binding that may occur through conjugation, cleavable linkers can also be used to mask the toxicity of the individual PAMPs until they reach their intended site, as has been illustrated in Mincle-NOD2 chimera studies [99]. Indeed, cleavable linkers have been successfully used for the preparation of other PAMP-containing bioconjugates, including a self-adjuvanting MDP–peptide–Pam_3_CSK_4_ conjugate [139]; hydrolysable antibody-drug conjugates containing resiquimod, which exhibited greater efficacy than those without a hydrolysable linker [140,141]; pH-sensitive linkers for the intracellular delivery of TLR7/8 agonists [142,143]; and redox-responsive disulfide linkers, which can be tuned to selectively favour the intracellular release of CpG [144]. Notwithstanding, the site of the cleavable linker still needs to be considered, as illustrated by Filippov and co-workers, who showed that the choice of conjugation site in their MDP–peptide–Pam_3_CSK_4_ conjugates had a marked impact on the ability of the conjugate to lead to cytokine production [139].

It is also interesting to note that the ability of the conjugates to form aggregates in solution may be positively correlated to their immunogenicity [80,81,82,103,105]. For example, the promising TLR2/6_TLR7/8 conjugate (**23**) [82] and TLR2_TLR7 (PamadiFectin, **19**) [80] spontaneously formed aggregates or were prepared as polymers, and this is thought to improve the therapeutic window while simultaneously improving the immune response to the conjugates [141]. This is also supported by earlier studies in which polymers containing multiple copies of imidazoquinoline-based small-molecule TLR7/8a agonists led to promising innate immune activation, as well as antibody and T cell responses [141]. The formation of aggregates may also enhance endocytosis, which, in turn, may lead to more effective endosomal PRR signalling, as seen in studies by Laiño et al., with TLR2-mediated endocytosis and subsequent TLR7 activation [81], and by Ding et al., with TLR4-mediated endocytosis being proposed to enhance NOD2 signalling [103].

**Figure 12:**
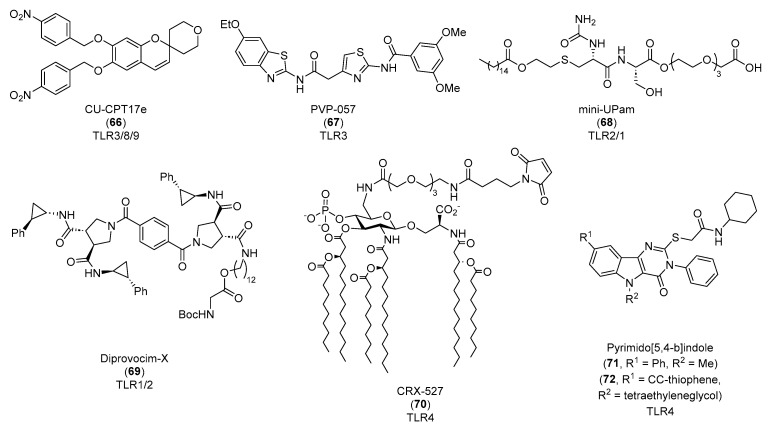
Other PAMPs that could be used for conjugate synthesis.

In addition to the PAMPs used in PAMP-PAMP conjugates to date, there exist many other possible combinations of ligands that could be used [24,119,145,146]. These include agonists for hitherto unexplored PAMP chimeras targeting TLR3 and/or TLR5, which recognise relatively large and structurally constrained ligands, making them less amenable to the types of synthetic conjugation strategies commonly used to generate dual-PAMP agonists. TLR3 signalling depends on dsRNA length, duplex integrity, and receptor dimerization along the RNA backbone [27], and introducing linkers, bulky substituents, or additional PAMPs into the dsRNA can disrupt these requirements. Similarly, TLR5 recognition involves a specific protein–protein interaction between flagellin and the receptor ectodomain [147], with covalent modification of flagellin again risking perturbing folding or masking receptor-binding surfaces. In addition, producing homogeneous, site-specific conjugates involving RNA or recombinant proteins is substantially more difficult than synthesising small-molecule or lipopeptide conjugates and has led researchers to often favour co-formulation, nanoparticle co-encapsulation, or fusion protein approaches rather than the development of true TLR3 or TLR5 covalent chimeras. Notwithstanding, several ligands, such as CU-CPT17e (**66**, Figure 12), which is capable of simultaneously activating TLRs 3, 8, and 9 [148], and PVP-057 (**67**) [149], which has shown promising TLR3 adjuvant activity, have been identified. However, as noted above, conjugation may limit their utility, thereby necessitating strategies where the ligands are released in the endosome to allow for effective TLR3 activation. Alternatively, phosphoramidate chemistry could be employed to allow for the selective modification of the TLR3 ligand poly(I:C) at its terminus [28,150].

With regard to the PAMPs that have already been targeted using existing PAMP chimeras, alternative ligands include the TLR2/1 agonists mini-Upam (**68**), which is less lipophilic and therefore easier to formulate [151], and Diprovocim-X (**69**), which was optimised from SAR studies and contains a bioconjugation handle some distance from the TLR binding site [152,153]. TLR4 agonist CRX-527 (**70**) is an alternative to MPL, with SAR optimisation leading to the development of derivatives with a maleamide-terminated PEG linker, which has been used for successful conjugation to antigens [154,155]. Modifications to the pyrimido[5,4-*b*]indoles also suggest some further analogues that may show improved TLR4 activity, including indole *N*-methylation (e.g., **71**) [52] or thiophene derivative (**72**) [156], which contains the conjugation handle from the indole. Efforts towards the development of small-molecule TLR9 ligands are ongoing; however, the only currently identified small molecules targeting TLR9 act as antagonists rather than agonists [146].

Multiple PAMPs could also be combined with antigens to create self-adjuvanting vaccines, as illustrated by a DC-SIGN-targeted trifunctional conjugate that uses the multivalent presentation of CLR ligands on tumour antigens together with a TLR7 agonist to induce tumour-specific T cells [139,157], or they could be combined with other immunomodulatory compounds, as illustrated by the MDP_Pam_2_CSK_4__peptide conjugate that activated NOD2, TLR2/6 and the inflammasome [158]. Finally, PAMP chimeras may also hold much promise in the development of trained immunity [159]. This is particularly relevant given the speed at which pathogens are now transmitted globally and the potential need for therapies that may offer some protection while vaccines targeting adaptive immunity are developed.

## 6. Conclusions

Adjuvants are important components of vaccines, and the development of PAMP conjugates, which activate multiple PRRs, represents a promising new avenue for adjuvant research. PAMP conjugates can induce downstream signalling pathways and cytokine and chemokine secretion to initiate an adaptive immune response and immunological memory. Moreover, the concomitant activation of different PRR pathways by PAMP conjugates can result in an immune response that is greater than that elicited by the individual PAMPs. PAMP chimeras can also prevent systemic inflammation that is caused by one or more of the admixed PAMPs, while PAMPs for extracellular PRRs may function as “targeting” ligands that direct the construct to certain cell populations where cellular uptake is then enhanced via endocytosis.

With these advantages in mind, it is therefore not surprising that several PAMP chimeras exhibit particularly promising adjuvant activity, including those containing TLR (e.g., Pam_2_Cys) or NOD2 (e.g., murabutide or MDP) ligands in combination with other PAMPs. Insight into the potential role of aggregation, endosomal targeting, and the overall effect of linker/PAMP molecular weight on PRR signalling, and therefore immune cell activation in response to the PAMP conjugate, has been provided. However, it is still not possible to predict with certainty how different PAMPs should be conjugated to elicit the desired immune response, and not all PAMP conjugates led to an immune response that is greater than that of the admixed PAMPs. Accordingly, further studies into PAMP conjugates will not only aid in the development of more effective adjuvants for antigen-specific vaccines, but these conjugates could also be used to instigate trained immunity or as mechanistic tools to contribute to our understanding of signalling interactions between PRR pathways and PRR synergy.

## Figures and Tables

**Table 1:** TLR ligand conjugates.

Comp.	Ligand 1	Ligand 2	Study	Results	Reference
**16**	Pam_3_CSK_4_ (**4**) (TLR2/1)	Indole (**8b**) (TLR4)	Conjugate vs. admix or single PAMP; kinetics	Chimera: NF-κB↓, IL-6↓, TNF↓ (Mφ), cf. admix; mainly TLR4 signalling.	Kimani et al. (2021) [78]
**17**	Pam_2_CSK_4_ (**2**) (TLR2/6)	Indole (**8b**) (TLR4)	Chimera vs. admix or single PAMP; kinetics	Chimera: NF-κB↓, IL-6↓, TNF↑, no additive effect or synergy.	Kimani et al. (2021) [78]
**18**	CU-T12-9 (**6**) (TLR2/1)	Indole (**8c**) (TLR4)	Chimera vs. admix	No added effect (hPBMC).	Janež et al. (2025) [79]
**19**	Pam_2_Cys (**1**) (TLR2/6)	CL307 (**11b**) (TLR7)	Chimera vs. admix or single PAMP; adjuvanticity (HIV-1 p24 antigen)	Chimera: DC maturation↑, transcription↑. CD8^+^T cell priming: Similar to admix; Ab avidity↑, balanced T_H_1/T_H_2, less toxicity.	Gutjahr et al. (2017) [80]
**20**	Pam_2_Cys (**1**) (TLR2/6)	CL307 (**11b**) (TLR7) [No linker]	Chimera vs. admix or single PAMP; in vitro DC, T cell, mast cell activation	Chimera: In vitro: IL-1ß↓, IL-6↓, enhanced OVA-induced IL-17A, mast cell activation (limited); in vivo cytokines (-).	Laiño et al. (2017) [81]
**21**	Pam_2_CSK_4_ (**2**) (TLR2/6)	CL307 (**11b**) (TLR7) [Linked to C-terminus]	Chimera vs. admix or single PAMP; DC, T cell, mast cell activation; adjuvanticity (allergy model)	Chimera: In vitro: IL-1ß↓, IL-10↑, suppressed OVA-induced IL-13, IL-5; mast cell activation (limited). In vivo: Cytokines↑ (<admix); IgG2a↑, IgE↓, ß-hexosaminidase↓.	Laiño et al. (2017) [81]
**22**	Pam_2_CSK_4_ (**2**) (TLR2/6)	CL307 (**11b**) (TLR7) [Linked to Lys-2]	Chimera vs. admix or single PAMP; DC, T cell, mast cell activation	Chimera: In vitro: IL-1ß↓, IL-6↑, IL-10↑, suppressed OVA-induced IL-13, IL-5; mast cell activation (limited); in vivo cytokines↑ (<admix).	Laiño et al. (2017) [81]
**23**	Pam_2_CSK_4_ (**2**) (TLR2/6)	Imidazoquinoline (**13a**) (TLR7/8) Cys-	Nanoassembly (carbohydrate polymer) vs. conjugate vs. admix; anticancer response	Nanoassembly > conjugate > admix: In vitro cytokines (Mφ), in vivo antitumour (B16F10 model), less toxicity.	Manna et al. (2020) [82]
**24**	Pam_3_CSK_4_ (**4**) (TLR2/1)	Imidazoquinoline (**13a**) (TLR7/8)	Chimera vs. admix or single PAMP; kinetics	Chimera: NF-κB↓, IL-6↓, TNF↓ (Mφ), cf. admix; no additive effect obs.	Kimani FW et al. (2021) [78]
**25**	Pam_2_CSK_4_ (**2**) (TLR2/6)	Imidazoquinoline (**13a**) (TLR7/8)	Chimera vs. admix or single PAMP; kinetics	Chimera: NF-κB↓, cf. admix, IL-6 and TNF similar to admix; no additive effect obs. (Mφ).	Kimani et al. (2021) [78]
**26**	CU-T12-9 (**6**) (TLR2/1)	Purine (**12**) (TLR7)	Chimera vs. admix	Enhanced cytokine release from human PBMCs, enhanced PBMC cytotoxicity.	Janež et al. (2025) [79]
**27**	LTA (**5**) (TLR2)	CpG_1826 (**15**) (TLR9)	Chimera vs. admix in vitro (Mφ and DCs); TLR2/TLR9 signalling	Chimera: NF-κB↑, DC activation (activation markers/cytokines↑). Signalling partially dependent on TLR2 and TLR9.	Mancini et al. (2014) [83]
**28** **a–c**	LTA (**5**) (TLR2)	CpG_1826 (**15**) (TLR9)	Length of PEG linker TLR2/TLR9 signalling	Chimera: NF-κB↑ (Mφ) (longer linker↑) IL-6↑ (BMDC) (linker no effect). Longer linker hindered TLR9 activation.	Ryu et al. (2016) [84]
**29** **a–c**	Pam_2_CSK_4_C (**2**) (TLR2/6) [Linked Cys]	CpG_1826 (**15**) (TLR9)	Length PEG linker Role of TLR2/TLR9 signalling	Chimera: No advantage over individual PAMPs. PEG_12_: NF-κB↓ (Mφ). Longer linker hindered TLR9 activation (but <LTA-CpG dimer).	Ryu et al. (2016) [84]
**30**	Pam_2_CSK_4_ (**2**) (TLR2/6) [Ala/N_3_-Lys]	CpG_1826 (**15**) (TLR9)	Chimera vs. admix or single PAMP; kinetics	Chimera: Slight NF-κB↑ (Mφ), no increase in cytokines cf. admix or individual PAMPs (BMDCs).	Kimani et al. (2021) [78]
**31**	Pam_3_CSK_4_ (**4**) (TLR2/1)	CpG_1826 (**15**) (TLR9)	Chimera vs. admix or single PAMP; kinetics	Chimera: NF-κB↑ (Mφ), TNF↑ (BMDCs) (low ligand concentration).	Kimani et al. (2021) [78]
**32**	Indole (**8c**) (TLR4)	Loxoribine (**10**) (TLR7)	Chimera vs. trimeric	Chimera: Mφ/DC activation↓. Transcription similar to resting DC.	Tom et al. (2015) [19]
**33**	Indole (**8c**) (TLR4)	Purine (**12**) (TLR7)	Chimera vs. admix	Broad immune activation in vitro; enhancement in Ag-specific responses in in vivo B16F10 tumour model.	Janež et al. (2025) [79]
**34**	Indole (**8c**) (TLR4)	CpG_1826 (**15**) (TLR9)	Chimera vs. trimeric	NF-κB (Mφ) and IL-12 expression similar to trimer (BMDC). High gene transcription (but less than that of trimer). Indole augmented CpG immune response.	Tom et al. (2015) [19]
**35**	Indole (**8b**) (TLR4)	CpG_1826 (**15**) (TLR9)	Length PEG linker Combination of TLR TLR4^−/−^ and TLR9^−/−^	Chimera: NF-κB↑ (Mφ), IL-6↑ (BMDC). PEG_12_: Greatest activity. Small size of indole cf. Pam_3_CSK_4_C or LTA had less effect on TLR9 signalling.	Ryu et al. (2016) [84]
**36**	Loxoribine (**10**) (TLR7)	CpG_1826 (**15**) (TLR9)	Chimera vs. trimeric	Chimera: NF-κB (Mφ), IL-12 and transcription↓ cf. trimer (BMDC); similar to CpG alone.	Tom et al. (2015) [19]

**Table 2:** Trimeric TLR ligands.

Comp.	Ligand 1	Ligand 2	Ligand 3	Study	Results	Reference
**37**	Pyrimido-indole (**8**) (TLR4)	Loxoribine (**10**) (TLR7)	CpG ODN (**15**) (TLR9)	Chimera vs. PAMPs (linked or singular); in vitro signalling; in vivo: adjuvant (smallpox)	Trimeric conjugate: NF-κB↑ (Mφ), IL-12↑ (BMDCs), T_H_1/T_H_2 gene transcription (BMDCs), MyD88 and TRIF signalling pathways; IgG1 antibody titres/breadth↑	Tom et al. (2015) [19]
**38**	Pyrimido-indole (**8**) (TLR4)	Loxoribine (**10**) (TLR7) Longer linker	CpG ODN (**15**) (TLR9)	Trimer vs. individual PAMPs Adjuvanticity: *Coxiella burnetii* (CBU-1910) then various antigens	NF-κB: similar (Mφ); interferon stimulatory genes (no change); T_H_1 cytokines↑ (BMDCs); in vivo: modest cytokines, weight loss Minimal Ab titres, T_H_1-skewed, IL-4 and IFN-γ (recall expt.) (CBU_1910); robust T_H_1-biased Ab responses, no weight loss or fever, partial protection (AddaVax + six *C. burnetii* Ags)	Albin et al. (2019) [86]; Gilkes et al. (2020) [87]
**39**	Pam_3_CSK_4_ (**4**) (TLR2/1)	Pyrimido-indole (**8**) (TLR4)	Imidazoquinoline (**13a**) (TLR7/8)	Trimer vs. individual PAMPs Adjuvanticity: *C. burnetii* (CBU-1910)	NF-κB:↓ (Mφ); interferon stimulatory genes↓ (Mφ); few cytokines (BMDMs); in vivo: very few cytokines, no weight loss Trimer weak Ab response cf. admix; IL-4 upon recall	Albin et al. (2019) [86] Gilkes et al. (2020) [87]
**40**	Pam_3_CSK_4_ (**4**) (TLR2/1)	Pyrimido-indole (**8**) (TLR4)	CpG ODN (**15**) (TLR9)	Trimer vs. individual PAMPs Adjuvanticity: *C. burnetii* (CBU-1910) then various antigens	NF-κB: similar (Mφ); interferon stimulatory genes↓ (Mφ); T_H_1 cytokines↑ (BMDCs); in vivo: modest cytokines (TNF, IL-6, IL-12) no weight loss Modest Ab response (similar to admix); IL-4 upon recall (using CBU-1910); robust T_H_1-biased Ab responses, partial protection (AddaVax + six *C. burnetii* Ags)	Albin et al. (2019) [86] Gilkes et al. (2020) [87]
**41**	Pam_2_CSK_4_ (**4**) (TLR2/6)	Pyrimido-indole (**8**) (TLR4)	Imidazoquinoline (**13**) (TLR7/8)	Trimer vs. individual PAMPs Adjuvanticity: *C. burnetii* (CBU-1910)	Trimer: NF-κB↓ (Mφ) where dimer of Pam_imidazole↑ activity; interferon stimulatory genes↓ (Mφ); T_H_2 cytokines↑ (BMDCs); in vivo: very few cytokines, no weight loss Good Ab response, slight T_H_2-skewing (no difference to admix group); challenge: less weight loss cf. admix; IL-4 upon recall; but no protection	Albin et al. (2019) [86] Gilkes et al. (2020) [87]
**42**	Pam_2_CSK_4_ (**4**) (TLR2/6)	Pyrimido-indole (**8**) (TLR4)	CpG ODN (**15**) (TLR9)	Trimer vs. individual PAMPs Adjuvanticity: *C. burnetii* (CBU-1910)	NF-κB: similar (Mφ); interferon stimulatory genes (no change) (Mφ); T_H_2 cytokines↑ (BMDCs); in vivo: modest cytokines (modest cytokines, no weight loss), weight loss Modest Ab response (similar to admix); IL-4 upon recall	Albin et al. (2019) [86] Gilkes et al. (2020) [87]

**Table 3:** Dimeric NOD2 and TLR agonists.

Comp.	NOD2 Ligand	TLR Ligand	Study	Results	Reference
**45**	MurabutideOBn (**43c**)	Pam_2_C (**1**) (TLR2/6)	In vitro maturation/activation of DC Adjuvanticity: Gag p24 (HIV-1) antigen Lethal mouse pneumovirus	Chimera: Signalled via TLR2 and NOD2 (HEK reporter cells). DC maturation (↑) and synergistic increase in cytokines compared to admix. IgG↑/IgA↑, balanced T_H_1/T_H_2, led to chemotaxis, no apparent toxicity. Conjugate prevents lethal infection; efficacy greater than admixed PAMPs.	Pavot et al. (2014) [53] Rice et al. (2016) [102]
**46**	Desmuramylpeptide (**44a**)	CU-T12-9 (**6**) (TLR2/1)	Chimera vs. admix	IL-6↑ (BMDC). Enrichment of IL-17 signalling pathway.	Janež et al. (2025) [79]
**47**	MDP (**43a**) Linked through Mur-C1	RC529 (**7**) (TLR4)	Chimera vs. admix and single PAMPs; IL-6 (in vitro and in vivo); signalling pathways; in vivo: IL-6; adjuvanticity (OVA)	Chimera: BMDCs: IL-6↑ via TLR4 and NOD2 (TLR4 impt for NOD2 activity); Mφ: CD80↑ CD86↑; in vivo: IL-6↑. As adjuvant (+OVA) *cf.* admix: IgG↑ (mainly IgG1); recall: TNFα↑, IFNγ↑, memory CD8^+^ T cell↑, also increase in CD4+ T cells (similar to admix); no toxicity.	Ding et al. (2024) [103]
**48**	Desmuramylpeptide (**44b**)	Indole (**8c**) (TLR4)	Chimera vs. admix	No added effect in vitro (hPBMCs).	Janež et al. (2025) [79]
**49**	Desmuramylpeptide (**44b**)	Pyrimido-indole (**8c**) (TLR4)	Linker chemistry Solubility; signalling, in vitro activation	Signal via NOD2, poor TLR4 signalling and cytokine production by BMDMs.	Paradiso et al. (2025) [104]
**49**	Desmuramylpeptide (**44b**)	AZ617 (**9**) (TLR4)	Linker chemistry Solubility; signalling, in vitro activation	Signal via NOD2 and modest TLR4 signalling, PEG-linked (three PEG motifs) and piperazine-linked modest cytokine production (hPBMCs); least soluble chimera had the greatest cytokine response.	Paradiso et al. (2025) [104]
**50**	MDP (**43a**)	CL239 (**11c**) (TLR7)	Comparison to admixed; adjuvanticity (NP-p24-HIV vaccine)	Chimera: Stable particles, signalled via NOD2 and TLR7; hPBMC activation (synergy cf. admix), cytokine production (synergy), autophagy↑, Memory CD8^+^ T cells↑. Adjuvanticity: Significant IgG↑/IgA↑, IFN-γ↑, IL-17; protection against vaccina virus expressing p24.	Gutjahr et. al. (2020) [105]
**51**	Desmuramylpeptide (**44b**) (metabolically more stable bis(2-aminoethyl)ether linker)	Purine-based agonist (**12**) (TLR7)	Comparison of linker (see below); signalling, cytokine production, T cell activation	Chimera: Signal TLR7 (good), NOD2 (poor); cytokines↑ > admix (hPBMCs and mixed lymphocyte assay), CD4^+^ and CD8^+^ T cell activation↑ (typically greater than admix for all readouts).	Guzelj et al. (2022) [101]
**52**	Desmuramylpeptide (**44b**) (cleavable ester linker)	Purine-based agonist (**12**) (TLR7)	Comparison of linker (see above); signalling, cytokine production, T cell activation; adjuvanticity (OVA)	Chimera: Signal via both TLR7 and NOD2 (comparable to individual PAMPs); cytokines↑ > admix (hPBMCs and mixed lymphocyte assay), CD4^+^ and CD8^+^ T cell activation↑ [typically greater than admix); IgG↑, IgG1↑, IgG2a↑ (not compared to admix).	Guzelj et al. (2022) [101]

**Table 4:** Other PAMP conjugates (RIG-I, Mincle and STING).

Comp.	PAMP 1	PAMP 2	Study	Results	Reference
**58**	RI (**53**) (RIG-I)	CU-T12-9 (**6**) (TLR2/1)	Conjugate vs. admix	Admix showed modest cytokine production in vitro (TNF, IL-6 and MCP-1, hPBMCs), with response greater than that elicited by conjugate	Janež et al. (2025) [79]
**59**	RI (**53**) (RIG-I)	Indole (**8c**) (TLR4)	Conjugate vs. admix	No significant cytokine production for admix or conjugate (hPBMCs)	Janež et al. (2025) [79]
**60**	RI (**53**) (RIG-I)	Purine (**12**) (TLR7)	Conjugate vs. admix; in vivo antitumour activity	Conjugate: in vitro, cytokines↑ cf. admix (hPBMCs; e.g., IL-12p70↑, IFN-γ↑, IP-10↑); conjugate led to (modest) reduction in tumour growth in vivo	Janež et al. (2025) [79]
**61**	RI (**53**) (RIG-I)	Desmuramylpeptide (**44b**) (NOD2)	Conjugate vs. admix	No significant cytokine production for admix (hPBMCs)	Janež et al. (2025) [79]
**62**	TDM (**54**) (Mincle)	MDP (**43a**) (NOD2)	Conjugate vs. single agonists	Conjugates activated murine Mφ to similar level as MDP alone and induced delayed-type hypersensitivity with same potency as TDM	Ishida et al. (1989) [107]
**63**	C18 Brar (**55**) (Mincle)	MDP (**43a**) (NOD2)	Conjugate vs. admix in vitro/in vivo; vaccine adjuvanticity	Conjugate: in vitro, no increase in cytokines (Mφ); enhanced antibody production of conjugate vs. admix in vivo	Dangerfield et al. (2024) [99]
**64**	C18Brar dilipid (**56**) (Mincle)	MDP (**43a**) (NOD2)	Conjugate vs. admix in vitro/in vivo; vaccine adjuvanticity	Conjugate: in vitro, cytokines↑ cf. admix (Mφ); in vivo, vaccination using conjugate enhanced memory recall and reduced toxicity	Dangerfield et al. (2024) [99]
**65**	CDN (**57**) (STING)	Pam_3_CSK_4_ (**4**) (TLR2/1)	In vivo anti-OVA antibody titres for conjugate vs. admix and in vivo antitumour immunity in B16-OVA tumour model	Conjugate induced significantly greater anti-body titres compared to admix and reduced tumour burden in B16-OVA tumour model	Hu et al. (2020) [108]

## Data Availability

No new data were created or analyzed in this study.

## Abbreviations

The following abbreviations are used in this manuscript:

Ab	Antibody
ADP	Adenosine diphosphate
AIM2	Absent in melanoma-2
ALR	AIM2-like receptor
AP-1	Activator protein 1
APC	Antigen-presenting cell
Bcl10	B-cell lymphoma/leukaemia 10
BMDC	Bone marrow-derived dendritic cell
BMDM	Bone marrow-derived macrophage
CARD	Caspase activation and recruitment domain
CARD9	Caspase recruitment domain-containing protein 9
CDN	Cyclic dinucleotide
cGAMP	Cyclic guanosine monophosphate–adenosine monophosphate
cGAS	Cyclic GMP-AMP synthase
CLR	C-type lectin receptor
CpG	Cytosine–phosphate–guanine
DC	Dendritic cell
ER	Endoplasmic reticulum
FcRγ	Fc receptor gamma
HEK	Human embryonic kidney
HIV	Human immunodeficiency virus
IFN	Interferon
Ig	Immunoglobulin
IL	Interleukin
IRF	Interferon regulatory factor
ISG	Interferon-stimulated gene
ITAM	Immunoreceptor tyrosine-based activation motif
LPS	Lipopolysaccharide
LRR	Leucine-rich repeat
LTA	Lipoteichoic acid
Malt1	Mucosa-associated lymphoid tissue lymphoma translocation protein 1
MAPK	Mitogen-activated protein kinase
MAVS	Mitochondrial antiviral signalling protein
MCP-1	Monocyte chemoattractant protein 1
MD-2	Myeloid differentiation factor 2
MDA5	Melanoma differentiation-associated protein 5
MDP	Muramyl dipeptide
MHC	Major histocompatibility complex
Mincle	Macrophage inducible C-type lectin
MTP	Muramyl tripeptide
MyD88	Myeloid differentiation factor 88
NF-κB	Nuclear factor kappa B
NHS	*N*-hydroxysuccinimide
NK	Natural killer
NLR	NOD-like receptor
NOD	Nucleotide-binding oligomerisation domain
NP-p24	Nanoparticle p24 antigen
ODN	Oligodeoxynucleotide
OVA	Ovalbumin
PAMP	Pathogen-associated molecular pattern
PBMC	Peripheral blood mononuclear cell
PBS	Phosphate-buffered saline
PEG	Polyethylene glycol
poly(I:C)	Polyinosinic–polycytidylic acid
PRM	Pattern-recognition molecule
PRR	Pattern-recognition receptor
RIG-I	Retinoic acid-inducible gene-I
RIPK2	Receptor-interacting serine/threonine-protein kinase 2
RLR	RIG-I-like receptor
SAR	Structure–activity relationship
SLP	Synthetic long peptide
STING	Stimulator of interferon genes
Syk	Spleen tyrosine kinase
TDB	Trehalose dibehenate
TDM	Trehalose dimycolate
TH	T-helper
TIR	Toll/interleukin-1 receptor
TLR	Toll-like receptor
TNF	Tumour necrosis factor
TRIF	TIR-domain-containing adaptor inducing interferon-β
TRM	Tissue-resident memory T cell
